# CO-to-sugars conversion from one-pot two-step electro-organocatalytic process

**DOI:** 10.1039/d5sc06667k

**Published:** 2025-10-23

**Authors:** Ajeet Singh, David Martins-Bessa, Julien Bonin, Marc Robert, Sébastien Bontemps

**Affiliations:** a Institut Parisien de Chimie Moléculaire (IPCM), Sorbonne Université, CNRS F-75005 Paris France julien.bonin@sorbonne-universite.fr marc.robert@sorbonne-universite.fr; b Laboratoire de Chimie de Coordination (LCC), Université de Toulouse, CNRS F-31077 Toulouse Cedex 04 France sebastien.bontemps@lcc-toulouse.fr; c Institut Universitaire de France (IUF) F-75005 Paris France

## Abstract

The conversion of C_1_ molecules (single-carbon species) into C_*n*_ products (carbon chains) is a key challenge for developing sustainable chemical feedstocks to replace fossil resources. Carbohydrates, a vital class of complex polycarbon molecules, are mainly extracted from biomass, but *de novo* synthesis provides a complementary route to access rare and non-natural carbohydrates. Here, we report a fully integrated, one-pot two-step system converting carbon monoxide (CO) into carbohydrates. This process couples the electroreduction of CO to formaldehyde with the organocatalytic oligomerization of formaldehyde into C_5–6_ carbohydrates selectively. This work establishes a novel pathway to utilize CO as a building block for synthesizing complex carbon chains.

## Abstract 2

A one-pot two-step process combining electroreduction and organocatalysis to selectively transform CO into C_5–6_ carbohydrates with formaldehyde as the key intermediate.

## Graphical abstract

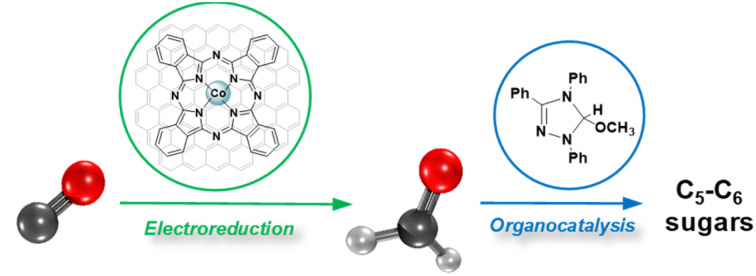

## Introduction

Carbon monoxide (CO) has been present in trace amounts in Earth's atmosphere since ancient times^1^ and plays an important role in various biological processes.^2^ It has also been detected both in outer space and in the atmosphere of other planets.^3^ For decades, CO has been a pivotal molecule in chemistry, serving as a key ligand in transition metal complexes^4–6^ and as a vital feedstock for large-scale industrial processes such as hydroformylation, the Monsanto process, and the Fischer–Tropsch synthesis.^7,8^ More recently, CO has also attracted significant interest in main group chemistry.^9,10^ Current industrial production of CO relies on carbon-intensive processes like coal gasification, steam reforming of natural gas and partial oxidation of hydrocarbons.^3^ These fossil-based routes have recently been supplemented by newer synthetic methods: biomass conversion^11–13^ and CO_2_ reduction. CO_2_ reduction strategies mainly include hydrogenation (*via* the water-gas shift reaction),^14,15^ and electrochemical reduction. Notably, efficient electrochemical CO_2_-to-CO conversion – enabled by nanomaterials or transition metal catalysts – represents significant advances nearing maturity at the laboratory scale due to improved mechanistic understanding, with industrial-scale development now imminent.^16,17^

**Scheme 1:**
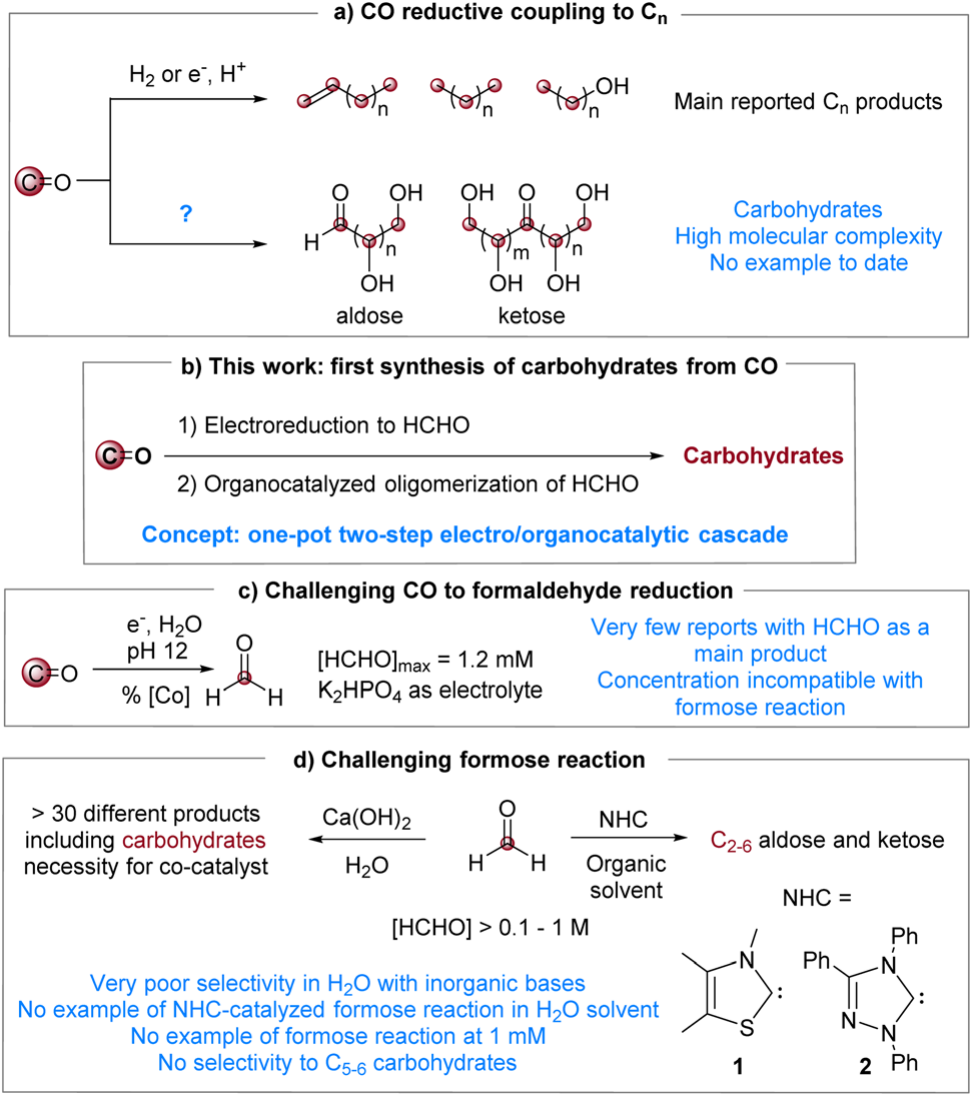
(a) CO conversion to C_*n*_ compounds; (b) overview of the present work; (c) status and challenges of CO to HCHO electro-conversion; (d) status and challenges of formose reaction catalyzed by inorganic base (in H_2_O) and by NHC (in organic solvent).

These sustainable pathways have amplified interest in CO utilization as a single-carbon (C_1_) synthon for generating carbon chains (C_2+_ products) for applications as energy carriers or chemical feedstocks.^18–20^ Nevertheless, CO-derived products remain predominantly limited to highly reduced compounds – primarily hydrocarbons, aliphatic alcohols, and olefins – across most reported systems (Scheme 1a). The two principal reductive pathways are (i) high-temperature/pressure CO hydrogenation (*via* Fischer–Tropsch synthesis with syngas, *i.e.* a CO/H_2_ mixture), the oldest industrial process for converting C_1_ to C_*n*_ products and (ii) electrochemical CO reduction, which yields similar compounds under milder conditions but with shorter chain lengths.^21–23^ CO electroreduction products are indeed usually limited to C_2_ and C_3_ chains, mainly employing Cu-based catalysts,^24,25^ although recent results have shown that this “short chain wall” could be broken with Ni^26,27^ and Au/Ni^28^ systems notably, to generate C_3_–C_7_ hydrocarbons or α-olefins. These reduced products exhibit high energy density and substantial value as chemical feedstocks compared to C_1_ molecules. Nevertheless, less reduced polyoxygenated compounds would offer greater chemical complexity and broader synthetic versatility. Such molecules – particularly long-chain C_2+_ polyoxygenated products – remain rarely synthesized or even observed in CO transformations.^24,25,29,30^ This feature is explained by the easy deoxygenation of the reaction intermediates before or after C–C bond formation under the applied conditions. The over-reduction event thus prevents the accumulation of polyoxygenated products.

Carbohydrates are a class of polyoxygenated compounds which is ubiquitous in natural processes, because their molecular complexity is used as ideal key/lock tools in biological recognition. Besides the extraction of the naturally abundant carbohydrates from biomass and their use as feedstock in further – often biocatalyzed – transformations, there is a strong long-term interest in the *de novo* synthesis of less abundant or non-natural species from non-natural resources.^31,32^ In this domain, a new emerging field aims at using sustainable C_1_ source as the only source of carbon. CO_2_ was the obvious first explored molecule for this purpose. Only a few examples have been reported so far, underscoring the challenges of (i) integrating multiple steps into fully operational systems, (ii) achieving atom-economical transformations and (iii) operating under mild conditions.^31,32^ In none of these cases does CO act as an intermediate. Consequently, developing methods to synthesize carbohydrates directly from CO would establish both a novel route to carbohydrates and an innovative pathway for CO utilization as a multi-carbon building block (Scheme 1a).

To prevent over-reduction, our approach centers on accumulating formaldehyde from CO reduction as a critical first step, followed by controlled C–C coupling to ultimately generate carbohydrates. The oligomerization of formaldehyde – the formose reaction – is indeed a reaction that gives rise to carbohydrates. However, CO-to-formaldehyde reduction remains underexplored,^33–36^ while achieving selective formose reaction is significantly challenging in particular in aqueous conditions.^37^ In this work, we report an integrated electro/organocatalytic system for converting CO to C_5–6_ sugars under mild conditions. The sequence process is not merely an extension of C–C coupling strategies used for producing hydrocarbons, but opens a new conceptual pathway for sustainable synthesis of sugars, a feat that biochemistry typically accomplishes *via* highly evolved enzyme cascade. Our one-pot two-step approach combines (i) the electrocatalytic CO-to-formaldehyde reduction using a molecular cobalt catalyst and (ii) the organocatalyzed formose transformation in the same aqueous electrolyte with triazolium-based catalyst (Scheme 1b). Overcoming three key challenges – inherent difficulties in each step plus their synergistic integration – our approach unlocks this unprecedented transformation.

There are indeed limited reports of CO reduction to formaldehyde.^33–35^ We recently demonstrated that the electroreduction of CO in aqueous solution at pH 12, using potassium phosphate as electrolyte and a Co-based molecular catalyst, generates formaldehyde, along with methanol and hydrogen as a by-products.^33^ A maximum formaldehyde concentration of 1.2 mM was obtained after 30 min, which represents the highest reported one for such transformation (Scheme 1c).

The formose reaction – first documented in the 19th century^38^ – typically yields complex mixtures containing carbohydrates, carboxylic acids, and polyols.^37^ Product distribution proves highly sensitive to reaction conditions (notably time and solvent) and catalytic systems. While inorganic bases – such as Ca(OH)_2_ – can catalyze the reaction, their inability to promote formaldehyde dimerization in solution^39^ necessitates co-catalysts and typically generates mixtures of up to 30 products (Scheme 1d).^37^ The utilization of specific N-Heterocyclic Carbene (NHC), such as thiazolium- and triazolium-based compound 1 and 2 (Scheme 1d), able to notably catalyze the dimerization of formaldehyde by Umpolung, have been shown to improve the selectivity of this transformation in organic solvent.^40,41^ Thiazolium-based catalysts are inactive in pure water^42^ and display only moderate activity when limited amounts of water are present in organic solvents.^43^ Although we demonstrated that triazolium-based catalysts can withstand up to 10% water in THF during the formose reaction, selectively producing glycolaldehyde (a C_2_ carbohydrate),^44^ their application under fully aqueous conditions has not yet been reported. Likewise, no study has described the formose reaction at formaldehyde concentrations as low as 1 mM. We therefore focused on formaldehyde oligomerization, with particular attention to low-concentration conditions.

## Results and discussion

### Formose reaction in aqueous media: NHC catalysis and concentration limits

NHC 1 and 2 were evaluated under rather standard conditions, *i.e.* 0.5 mol% catalyst loading, 80 °C, and 30 min (Table 1).^41,44^ Compound 3 was also tested because of its *in situ* formation from the reaction of 2 with methanol – a component present in the electroreduction mixture. Additionally, our prior work demonstrated that 3 achieves comparable performance to 2 in the formose reaction within a THF/H_2_O mixture, selectively yielding glycolaldehyde.^44^ Moreover, compound 3 being air stable contrarily to 1 and 2, it does not require inert conditions during storage and handling. The initial exploration showed that although 1 does not catalyze the reaction (Table 1, entries 1 and 5), given its known instability in H_2_O,^45^ compounds 2 and 3 catalyze the reaction to C_2–6_ carbohydrates with high yields of 82% and 71%, respectively, at [HCHO] = 1 M (Table 1, entries 2–3). In the absence of catalyst, no carbohydrate was detected (Table 1, entry 4), confirming KOH inability to catalyze the reaction under these conditions. Furthermore, adding 18-crown-6 (1 : 1 to 0.01 M KOH) to coordinate K^+^ ions did not affect product formation with catalyst 2 (Fig. S14). This further confirms K^+^ negligible role in NHC catalysis, consistent with its minimal impact in the formose reaction compared to more influential cations like Ca^2+^.^46,47^ Formose reactions are conventionally conducted at concentrations exceeding 0.1–1 M, presumably because lower concentrations yield minimal or no carbohydrates.^41^ Given the maximum reported formaldehyde concentration from CO electroreduction is only 1.2 mM, we anticipated that concentrations would pose a significant challenge for our study. When catalysts 2 and 3 were tested at a formaldehyde concentration of 0.1 M, yields decreased to 31% and 26%, respectively (Table 1, entries 6–7). At even lower concentrations, *i.e.* 0.01 M or below, no carbohydrate was detected (Table 1, entries 8–10). pH optimization studies at 0.1 M revealed detrimental effects of pH 13 and 14 (Table 1, entries 11–12), likely due to the rapid disproportionation of formaldehyde to methanol and formic acid *via* the Cannizzaro reaction. In contrast, pH 8 afforded carbohydrates in 19% yield (Table 1, entry 13). Finally, the addition of an excess of MeOH (4 M) with catalysts 2 and 3 (Table 1, entries 14–15) did not modified the outcome of the catalysis.

**Table 1:** Initial exploration with NHC catalysts 1–3 for the formose reaction in aqueous solutions^a^

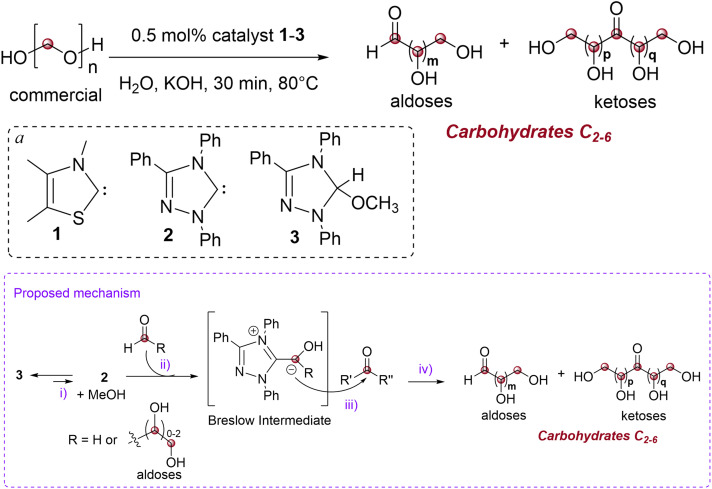					
Entries	[HCHO] (M)	pH	Catalyst^a^	C_2–4_	C_5–6_
1	1	12	1	nd	nd
2	1	12	2	35	47
3	1	12	3	41	30
4	1	12	**none**	nd	nd
5	0.1	12	1	nd	nd
6	0.1	12	2	5	26
7	0.1	12	3	5	21
8	0.01	12	2	nd	nd
9	0.01	12	3	1	nd
10	0.007	12	3	nd	nd
11	0.1	13	3	nd	nd
12	0.1	14	3	3	2
13	0.1	8	3	11	8
14^b^	0.1	12	2	5	24
15^b^	0.1	12	3	6	29

a See above.

b With the addition of 4 M of MeOH.

### Proposed mechanism

We propose that compound 3 generates carbene 2 through methanol elimination under the reaction conditions (Table 1, i), and that 2 serves as the active catalytic species for both compounds. A particularly notable feature of NHCs in organocatalysis is their capacity to induce Umpolung transformations of aldehydes. As first proposed by Breslow in 1958 and subsequently corroborated by Castells, Inoue, Teles, Enders, Tajima, and others,^40–42,48,49^ the carbene reacts with formaldehyde to form the elusive, yet crucial, Breslow intermediate (R =H, Table 1, ii). In this intermediate, the nucleophilic carbon atom of the former formaldehyde molecule can attack another molecule of formaldehyde (R, R′, R′′ = H, Table 1, iii), leading to the formation of glycolaldehyde, the C_2_ carbohydrate. A subsequent addition to a third formaldehyde molecule, followed by release of the carbene catalyst, yields the C_3_ carbohydrates glyceraldehyde or dihydroxyacetone. Alternatively, the Breslow intermediate (R=H, Table 1, ii) may react with the formed C_2_–C_5_ aldoses, accounting for the generation of C_3_–C_6_ aldoses. The formation of C_4_–C_6_ ketoses could be explained by Umpolung reactivity occurring not with formaldehyde, but with the produced C_2_–C_4_ aldoses (R(=CHOH)_0.2_–CH_2_OH, Table 1, ii). Although this mechanism is largely accepted, detailed experimental and theoretical studies of the NHC-catalyzed formose reaction accounting for the formation of the carbohydrates but also of other polyol chains (*vide infra*) remain scarce, most likely due to the complexity of the competing reactions involved in this process.

### Optimization of the formose reaction with catalyst 3

Compound 3 was selected to optimize carbohydrate formation at lower HCHO concentrations. Various reaction times and catalyst loadings were investigated (see Table S6 for the full list of tests), with the most significant results summarized in Table 2. Initial testing used 0.01 M HCHO. Despite extended reaction times (90–180 min) and higher catalyst loadings (5–20 mol%), only traces of carbohydrates were detected (Table 2, entry 1 and Table S6), although formaldehyde was fully consumed, forming unidentified products (likely carboxylic acids and polyols chains, *vide infra*).

**Table 2:** Optimized parameters and constraints, including pH, formaldehyde concentration and nature of the electrolyte, for the formose reaction using commercial HCHO and catalyst 3

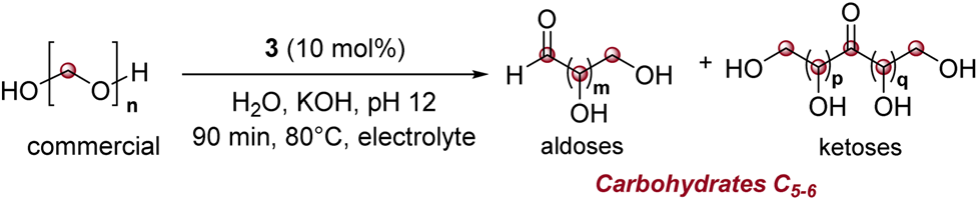				
Entries	[HCHO] (M)	Electrolyte	Yields (%)	
			C_2–4_	C_5–6_
1	0.01	—	2	0
2^a^	0.03	—	1	42
3	0.015	—	nd	2
4	0.019	—	nd	18
5	0.022	—	nd	18
6	0.026	—	1	21
7^b^	0.03	K_2_HPO_4_ or K_2_CO_3_	nd	nd
8^c^	0.03	KCI	nd	47

a Average yields over 9 runs, −13%/+10% deviation.

b [K_2_HPO_4_ or K_2_CO_3_] = 0.625 M.

c [KCI] = 1.3 M.

A formaldehyde concentration of 0.03 M was subsequently tested under similar conditions (Tables 2 and S6) and, encouragingly, carbohydrate formation was successfully achieved. Optimal conditions (90 min reaction, 10 mol% catalyst) established good yields in carbohydrates. The process demonstrated excellent reproducibility over three months across nine identical runs (Table S6). The average yield of C_2–6_ carbohydrates was 43%, with deviations ranging from −13% to +10% (Table 2, entry 2). The reaction exhibited high selectivity, yielding an average of 42% C_5–6_ carbohydrates and only 1% C_2–4_ carbohydrates. While we showed earlier that triazolium based compounds 2 and 3 catalyses the formose reaction in THF/H_2_O (10/1) to yield selectively glycolaldehyde (C_2_ carbohydrate), we show herein that the same catalyst can operate in water medium to achieve high selectivity for C_5–6_ chains without significant drop in yields. Interestingly, this C_5–6_ selectivity vanishes under identical conditions at elevated formaldehyde concentrations. The inherent complexity of the formose reaction prevented us from fully rationalizing this selectivity. We further systematically mapped the formaldehyde concentration threshold to generate carbohydrates. Trace carbohydrates (2% yield) emerged at 0.015 M, while at 0.019 M, 0.022 M and 0.026 M, carbohydrate yields of 18%, 18%, and 21% are obtained, respectively (Table 2, entries 3–6). These data establish a clear reaction threshold near 0.020 M under these conditions.

The electrolyte effect on the formose reaction was also investigated at 0.03 M HCHO solutions (Table S7). When potassium phosphate or potassium carbonate were used in electrolyte concentration (0.625 M), carbohydrate was undetectable in both cases (Table 2, entry 7). The 20-fold excess of these electrolytes compared to formaldehyde may inhibit the formose reaction itself^50^ or interfere with carbohydrate analysis indicating that electrolytes may pose significant compatibility challenges between electrocatalysis and other catalytic systems – a critical consideration for tandem one-pot reactions. Interestingly, KCl demonstrated full compatibility with the formose reaction: even at electrolyte concentrations (1.3 M), it afforded a 47% yield of exclusively C_5–6_ carbohydrates under otherwise identical conditions (Table 2, entry 8). Finally, the formose reaction was conducted with ^13^C-labeled formaldehyde. It not only confirmed that the observed carbohydrates arise from formaldehyde as the sole carbon source, but also that C_*n*_ chains other than carbohydrates are generated from the homocoupling of formaldehyde (Fig. S19 and Tables S9, S10).

### Electrolyte compatibility and formaldehyde concentration as key optimization parameters for CO electroreduction

As mentioned earlier, our recent work demonstrated electrochemical CO-to-HCHO conversion under controlled potential electrolysis (CPE) at *E*_electrode_ = −0.650 V *vs.* RHE (pH 12 phosphate buffer, *T* = 10 °C), formaldehyde (HCHO) and methanol (CH_3_OH) were observed. Notably, 30 min CPE yielded 1.2 mM HCHO (Table S12, entry 1).^33^ With the aim of increasing formaldehyde concentration, CPE duration was extended to 120 min leading to [HCHO] of 2.6 ± 0.6 mM (FE_HCHO_ = 15.6%; Fig. S22–S24). Critically, a three-compartment closed cell further boosted [HCHO] to 4 mM (Table S12, entry 2).

To ensure sufficient CO supply while preventing oxygen contamination, we implemented a continuous CO flow system. Key optimizations included extending electrolysis from 5 h to 10 h, increasing electrolyte volume from 5 mL to 12.5 mL (enhancing dissolved CO), and expanding electrode surface from 1 × 1 cm^2^ to 1.5 × 2 cm^2^ (larger electroactive surface). These modifications doubled HCHO production to 9.6 mM (Table S12, entry 4). Further extension to 12 h yielded 14.6 mM HCHO (Table S12, entry 5), confirming system stability beyond 10 h. Despite these gains, concentrations still remained insufficient for the formose reaction, and solvent evaporation attempts failed to increase the concentration. Progressively longer CPE durations achieved 17.4 mM HCHO after 15 h (Table S12, entry 6; Fig. S25 and S26) and 23.9 mM HCHO after 30 h (Table S12, entry 8; Fig. S27). Notably, no catalyst poisoning occurred at pH 12, in contrast with the report of HCHO induced deactivation at pH 6.8.^51^ However, beyond 30 h, pH rose to 13.3 despite buffering, accelerating Cannizzaro reactions. After 42 h, HCHO dropped to 7.8 mM with significant formate (HCOO^−^) formation (Fig. S28).

**Scheme 2:**
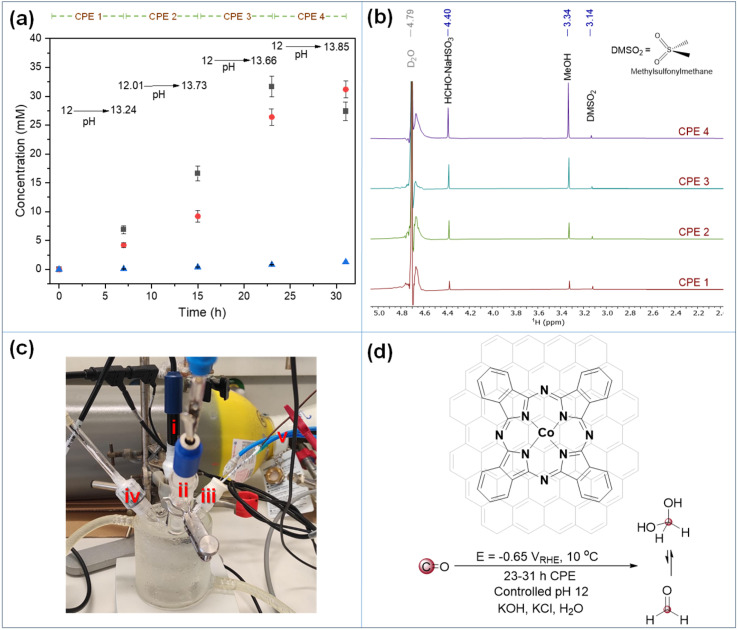
Electrochemical synthesis of formaldehyde (HCHO) from carbon monoxide (CO) gas. (a) Temporal concentration profiles of liquid-phase reduction products: HCHO (■), CH_3_OH (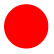) and HCOO^−^ (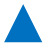) with pH variation indicated after each four successive CPE experiments (CPE 1–4, see ESI for details); (b) ^1^H NMR data after each CPE (CPE 1–4, from bottom to top; internal standard DMSO_2_ 0.33 mM); note that the HCOO^−^ peak is not shown due to its negligible production; expanded spectra are available in ESI, Fig. S31 and S32; (c) three compartment single cell including (i) glass pH electrode, (ii) saturated calomel electrode, (iii) CoPc/MWCNT working electrode, (iv) platinum counter electrode and (v) inlet and outlet of CO gas; (d) controlled potential electrolysis conditions for CO to formaldehyde using CoPc/MWCNT working electrode.

Finally, given the incompatibility of carbonate and phosphate electrolytes with formose reaction, we adopted a 1 M KOH electrolyte acidified to pH 12 with concentrated HCl (5 M) for optimized formaldehyde generation. The pH was adjusted every 7–8 hours during CPE (Scheme 2a). Although formaldehyde production was slower than in phosphate buffer, its concentration increased linearly (Fig. S29), reaching *ca.* 32 mM after 23 h (Scheme 2a and b). Subsequent CPE showed HCHO decline to 27.4 mM with concurrent methanol increase (from 26 to 31 mM), indicating onset of HCHO electroreduction. Reproducibility was demonstrated by achieving 29.4 mM HCHO after 23 h in a replicate experiment (Fig. S30).

### Proof-of-concept for a fully integrated one-pot two-step process

**Scheme 3:**
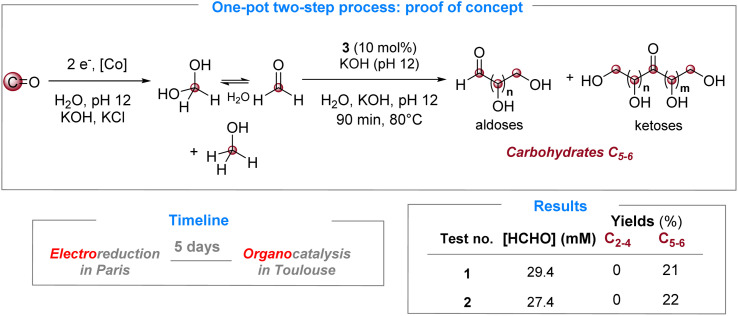
One-pot two-step transformation on the real system.

These electrolytic solutions of 27.4 and 29.4 mM obtained in Paris were then shipped to Toulouse to be tested in the formose reaction. Under optimized conditions (90 min, 80 °C, 10 mol% of 3), we were able to observe the formation of carbohydrates in both cases, with consistent yields of 21% and 22% for the 29.4 mM and 27.4 mM solutions, respectively (Scheme 3). These yields are in agreement with those produced with the commercial 26 mM formaldehyde solution (22%, Table 2, entry 6) and with the lower limit obtained with the 30 mM HCHO solution (30%, Table 2, entry 2). In addition, the selectivity to C_5–6_ carbohydrates was maintained like in the model reaction with commercial para-formaldehyde. The GC-MS analysis further revealed that non-carbohydrate C_2–6_ chains were also formed in the same mixture. These findings constitute the experimental proof of concept of the proposed CO-to-carbohydrate pathway and thus validate the optimization/compatibility studies described herein. Gaining deeper insight into the selectivity of the formose reaction under aqueous conditions, along with expanding the accessible formaldehyde concentration range, will further advance this promising approach for complex CO conversion and the *de novo* synthesis of carbohydrates.

## Conclusions

The conversion of CO to C_5–6_ carbohydrates in aqueous solutions was demonstrated with a fully integrated, one-pot two-step process. Our results establish that CO can be transformed into complex polyoxygenated products *via* formaldehyde as a key intermediate. The process integrates CO electroreduction to formaldehyde with subsequent organo-catalysed oligomerization of the resulting mixture into carbohydrates. Achieving compatibility between these steps was a significant challenge. By employing extended CPE duration, an enlarged electrode surface and pH control, we achieved formaldehyde concentrations approaching 30 mM, a thirtyfold increase compared to previous reports.^33–35^ However, such concentration was still low for the formose reaction, underscoring the difficulty of coupling electrocatalysis with organocatalysis. Despite the aqueous nature of the medium, the formose reaction was successfully conducted with a triazolium-based organocatalysts for the first time. Optimization at low formaldehyde concentration (30 mM) yielded an unprecedented C_5–6_ carbohydrates selectivity.

Conceptually, directly converting CO into carbohydrate backbones bypasses the need for traditional biomass oxidation routes or multistep, often enzyme-dependent, CO_2_-based pathways. Our strategy therefore introduces a new research direction focused on the direct electrochemical transformation of C_1_ compounds into sugars – a field that is still in its infancy with respect to selectivity, scalability, and energy efficiency.

## Author contributions

The manuscript was written through contributions of all authors. All authors have given approval to the final version of the manuscript. S. B., J. B. and M. R. conceived the idea and supervised the project. A. S. and D. M.-B. performed the experiments and measurements. All authors carried out data analysis, discussed the results and assisted with manuscript preparation.

## Conflicts of interest

There are no conflicts to declare.

## Supplementary Material

SC-016-D5SC06667K-s001

## Data Availability

Data are available upon reasonable request. Correspondence and requests should be addressed to julien.bonin@sorbonne-universite.fr; marc.robert@sorbonne-universite.fr; sebastien.bontemps@lcc-toulouse.fr. Supplementary information: experimental details on the preparation of catalytic electrodes; electrolysis experiments and analytical procedures (GC, GC-MS NMR) for detecting and quantifying reduction products and sugar products; additional cyclic voltammetry data and electrolysis experiments. See DOI: https://doi.org/10.1039/d5sc06667k.

## References

[cit1] Van Trump J. E., Miller S. L. (1973). Carbon monoxide on the primitive earth. Earth Planet. Sci. Lett..

[cit2] Piantadosi C. A. (2002). Biological Chemistry of Carbon Monoxide. Antioxid. Redox Signaling.

[cit3] Bierhals J. , in Ullmann's Encyclopedia of Industrial Chemistry, 2001, 10.1002/14356007.a05_203

[cit4] Bourissou D., Guerret O., Gabbaï F. P., Bertrand G. (2000). Stable Carbenes. Chem. Rev..

[cit5] Frenking G., Froehlich N. (2000). The Nature of the Bonding in Transition-Metal Compounds. Chem. Rev..

[cit6] Tolman C. A., Seidel W. C., Gosser L. W. (1974). Formation of three-coordinate nickel(0) complexes by phosphorus ligand dissociation from NiL_4_. J. Am. Chem. Soc..

[cit7] Fischer F. , Tropsch H., US Pat., US1746464A, 1930

[cit8] Peng J.-B., Geng H.-Q., Wu X.-F. (2019). The Chemistry of CO: Carbonylation. Chem.

[cit9] Fujimori S., Inoue S. (2022). Carbon Monoxide in Main-Group Chemistry. J. Am. Chem. Soc..

[cit10] Kong R. Y., Crimmin M. R. (2020). Cooperative strategies for CO homologation. Dalton Trans..

[cit11] Bolívar Caballero J. J., Zaini I. N., Yang W. (2022). Reforming processes for syngas production: a mini-review on the current status, challenges, and prospects for biomass conversion to fuels. Applications in Energy and Combustion Science.

[cit12] Gao Y., Wang M., Raheem A., Wang F., Wei J., Xu D., Song X., Bao W., Huang A., Zhang S., Zhang H. (2023). Syngas Production from Biomass Gasification: Influences of Feedstock Properties, Reactor Type, and Reaction Parameters. ACS Omega.

[cit13] Müller F. J., Fuchs J., Fanjul Cuesta M., Oblanca Gutiérrez A., Pratschner S., Müller S., Winter F. (2024). CO_2_ conversion to CO by fluidized bed biomass gasification: analysis of operational parameters. J. CO_2_ Util..

[cit14] González-Castaño M., Dorneanu B., Arellano-García H. (2021). The reverse water gas shift reaction: a process systems engineering perspective. React. Chem. Eng..

[cit15] Patel R., Varatharajan P., Zhang Q., Li Z., Gu S. (2025). Catalysts in the water-gas shift reaction: a comparative review of industrial and academic contributions. Carbon Capture Sci. Technol..

[cit16] Du S., Yang P., Li M., Tao L., Wang S., Liu Z.-Q. (2024). Catalysts and electrolyzers for the electrochemical CO_2_ reduction reaction: from laboratory to industrial applications. Chem. Commun..

[cit17] Jin S., Hao Z., Zhang K., Yan Z., Chen J. (2021). Advances and Challenges for the Electrochemical Reduction of CO_2_ to CO: From Fundamentals to Industrialization. Angew. Chem., Int. Ed..

[cit18] Fors S. A., Malapit C. A. (2023). Homogeneous Catalysis for the Conversion of CO_2_, CO, CH_3_OH, and CH_4_ to C_2+_ Chemicals via C–C Bond Formation. ACS Catal..

[cit19] Liang H.-Q., Beweries T., Francke R., Beller M. (2022). Molecular Catalysts for the Reductive Homocoupling of CO_2_ towards C_2+_ Compounds. Angew. Chem., Int. Ed..

[cit20] Liu Y., Deng D., Bao X. (2020). Catalysis for Selected C1 Chemistry. Chem.

[cit21] Guan Y., Li Y., Li Z., Hou Y., Lei L., Yang B. (2025). Promotion of C–C Coupling in the CO_2_ Electrochemical Reduction to Valuable C_2+_ Products: From Micro-Foundation to Macro-Application. Adv. Mater..

[cit22] Guo S., Wang J., Zhang H., Iloeje C. O., Liu D.-J. (2025). Direct Electrochemical Reduction of CO_2_ to C_2+_ Chemicals: Catalysts, Microenvironments, and Mechanistic Understanding. ACS Energy Lett..

[cit23] Tran N. H., Schreiber M. W., Fontecave M. (2025). Catalysts for selective CO_2_/CO electroreduction to C_3+_ compounds. EES Catal..

[cit24] Ruqia B., Tomboc G. M., Kwon T., Kundu J., Kim J. Y., Lee K., Choi S.-I. (2022). Recent advances in the electrochemical CO reduction reaction towards highly selective formation of C_x_ products (X = 1–3). Chem Catal..

[cit25] Zheng Y., Vasileff A., Zhou X., Jiao Y., Jaroniec M., Qiao S.-Z. (2019). Understanding the Roadmap for Electrochemical Reduction of CO_2_ to Multi-Carbon Oxygenates and Hydrocarbons on Copper-Based Catalysts. J. Am. Chem. Soc..

[cit26] Liu D.-J. (2022). Electrochemical conversion of CO_2_ to long-chain hydrocarbons. Joule.

[cit27] Zhou Y., Martín A. J., Dattila F., Xi S., López N., Pérez-Ramírez J., Yeo B. S. (2022). Long-chain hydrocarbons by CO_2_ electroreduction using polarized nickel catalysts. Nat. Catal..

[cit28] Kim Y. J., Maeng J. Y., Hwang S. Y., Yang J. H., Yoon I., Myung C. W., Rhee C. K., Sohn Y. (2023). Unlocking long-chain hydrocarbons (C_2–7_) via direct electrochemical CO_2_ and CO reduction on balanced Au/Ni electrodes. Nano Energy.

[cit29] Bertheussen E., Verdaguer-Casadevall A., Ravasio D., Montoya J. H., Trimarco D. B., Roy C., Meier S., Wendland J., Nørskov J. K., Stephens I. E. L., Chorkendorff I. (2016). Acetaldehyde as an Intermediate in the Electroreduction of Carbon Monoxide to Ethanol on Oxide-Derived Copper. Angew. Chem., Int. Ed..

[cit30] Kim D., Kley C. S., Li Y., Yang P. (2017). Copper nanoparticle ensembles for selective electroreduction of CO_2_ to C_2_-C_3_ products. Proc. Natl. Acad. Sci. U. S. A..

[cit31] Desmons S., Grayson-Steel K., Nuñez-Dallos N., Vendier L., Hurtado J., Clapés P., Fauré R., Dumon C., Bontemps S. (2021). Enantioselective Reductive Oligomerization of Carbon Dioxide into L-Erythrulose via a Chemoenzymatic Catalysis. J. Am. Chem. Soc..

[cit32] (d) Yang P. , Luo J. and Soland N.E., WO Pat., WO2025085698A1, 2025

[cit33] Singh A., Zamader A., Khakpour R., Laasonen K., Busch M., Robert M. (2024). Molecular Electrochemical Catalysis of CO-to-Formaldehyde Conversion with a Cobalt Complex. J. Am. Chem. Soc..

[cit34] Boutin E., Wang M., Lin J. C., Mesnage M., Mendoza D., Lassalle-Kaiser B., Hahn C., Jaramillo T. F., Robert M. (2019). Aqueous Electrochemical Reduction of Carbon Dioxide and Carbon Monoxide into Methanol with Cobalt Phthalocyanine. Angew. Chem., Int. Ed..

[cit35] Chatterjee T., Boutin E., Robert M. (2020). Manifesto for the routine use of NMR for the liquid product analysis of aqueous CO_2_ reduction: from comprehensive chemical shift data to formaldehyde quantification in water. Dalton Trans..

[cit36] Desmons S., Bonin J., Robert M., Bontemps S. (2024). Catalytic reduction of CO_2_ with 4e: formaldehyde, acetal synthesis and complex transformations. Chem. Sci..

[cit37] Delidovich I. V., Simonov A. N., Taran O. P., Parmon V. N. (2014). Catalytic Formation of Monosaccharides: From the Formose Reaction towards Selective Synthesis. ChemSusChem.

[cit38] Butlerow A. (1861). Formation synthétique d'une substance sucrée. Comptes rendus de l'Académie des Sciences.

[cit39] Eckhardt A. K., Linden M. M., Wende R. C., Bernhardt B., Schreiner P. R. (2018). Gas-phase sugar formation using hydroxymethylene as the reactive formaldehyde isomer. Nat. Chem..

[cit40] Castells J., Geijo F., López-Calahorra F. (1980). The “formoin reaction”: a promising entry to carbohydrates from formaldehyde. Tetrahedron Lett..

[cit41] Henrique Teles J., Melder J.-P., Ebel K., Schneider R., Gehrer E., Harder W., Brode S., Enders D., Breuer K., Raabe G. (1996). The chemistry of stable carbenes. Part 2. Benzoin-type condensations of formaldehyde catalyzed by stable carbenes. Helv. Chim. Acta.

[cit42] Matsumoto T., Yamamoto H., Inoue S. (1984). Selective Formation of Triose from Formaldehyde Catalyzed by Thiazolium Salt. J. Am. Chem. Soc..

[cit43] Shigemasa Y., Ueda T., Sashiwa H., Saimoto H. (1991). Formose Reactions. XXXI. Synthesis of Dl-2-C-Hydroxymethyl-3-Pentulose from Formaldehyde in N,N-Dimethylformamide-Water Mixed Solvent (I). J. Carbohydr. Chem..

[cit44] Zhang D., Jarava-Barrera C., Bontemps S. (2021). Selective Reductive Dimerization of CO_2_ into Glycolaldehyde. ACS Catal..

[cit45] Hollóczki O., Kelemen Z., Nyulászi L. (2012). On the Organocatalytic Activity of N-Heterocyclic Carbenes: Role of Sulfur in Thiamine. J. Org. Chem..

[cit46] Omran A., Menor-Salvan C., Springsteen G., Pasek M. (2020). The Messy Alkaline Formose Reaction and Its Link to Metabolism. Life.

[cit47] Waki M., Shirai S., Hase Y. (2024). Saccharide formation by sustainable formose reaction using heterogeneous zeolite catalysts. Dalton Trans..

[cit48] Tajima H., Inoue H., Ito M. M. (2003). A Computational Study on the Mechanism of the Formose Reaction Catalyzed by the Thiazolium Salt. J. Comput. Chem., Jpn..

[cit49] Desmons S., Fauré R., Bontemps S. (2019). Formaldehyde as a Promising C_1_ Source: The Instrumental Role of Biocatalysis for Stereocontrolled Reactions. ACS Catal..

[cit50] Wang N., Kong Y., Li J., Hu Y., Li X., Jiang S., Dong C. (2022). Synthesis and application of phosphorylated saccharides in researching carbohydrate-based drugs. Bioorg. Med. Chem..

[cit51] Yu S., Yamauchi H., Menga D., Wang S., Herzog A., Xu H., Zheng D. J., Wang X., Iriawan H., Huang B., Nitsche A., Shao-Horn Y. (2025). Reactivating Molecular Cobalt Catalysts for Electrochemical CO_2_ Conversion to Methanol. J. Am. Chem. Soc..

