# Supplementary material for: CO-to-sugars conversion from one-pot two-step electro-organocatalytic process
Source: Chem Sci. 2025 Oct 23;16(48):22996–3004. doi: 10.1039/d5sc06667k (PMC12593832; doi:10.1039/d5sc06667k)
Supplement: SC-016-D5SC06667K-s001 [file SC-016-D5SC06667K-s001.pdf]

Supplementary Information (SI) for Chemical Science.
This journal is © The Royal Society of Chemistry 2025

Electronic Supporting Information

CO-to-sugars conversion from one-pot two-step electro-organocatalytic process

Ajeet Singh,[a†] David Martins-Bessa,[b†] Julien Bonin,*[a] Marc Robert,*[a,c] and Sébastien Bontemps*[b]

[a] Dr. A. Singh, Prof. Dr. J. Bonin, Prof. Dr. M. Robert
Institut Parisien de Chimie Moléculaire (IPCM), Sorbonne Université, CNRS, F-75005 Paris, France
E-mail: julien.bonin@sorbonne-universite.fr, marc.robert@sorbonne-universite.fr

[b] D. Martins-Bessa, Dr. S. Bontemps
Laboratoire de Chimie de Coordination (LCC), Université de Toulouse, CNRS, F-31077 Toulouse Cedex 04, France
E-mail: sebastien.bontemps@lcc-toulouse.fr

[c] Institut Universitaire de France (IUF), F-75005 Paris, France

Table of contents

1. Materials 3
1.1 Chemicals 3
1.2 Procedures 3
1.3 CoPc/MWCNT film preparation 3
1.4 Electrolyte preparation....3
1.5 Stock solution for the formose reaction....4
2. Methods 4
2.1 General procedure for reaction with commercial paraformaldehyde 4
2.2 General method of controlled potential electrolysis....5
2.3 Final optimized procedure to electrochemically accumulate higher amounts of HCHO....5
2.4 Method of formaldehyde and methanol quantification 5
2.5 Method of characterization of carbohydrates 6
2.5.1 Derivatization and GC/MS analysis 6
2.5.2 Calibration curves....8
2.5.3 Yield calculation....12
2.5.4 Detailed calculation on one example....12
3. Formose reaction with commercial paraformaldehyde 15
3.1 Initial evaluation of the formose reaction catalyzed by **1-3** at 1, 0.1 and 0.01 M HCHO....15
3.2 Influence of the pH....17
3.3 Effect of the potassium cation 17
3.4 HCHO concentration, reaction time and catalyst loading....18
3.5 HCHO consumption 19
3.6 Impact of the electrolyte 21
4. Formose reaction with commercial ^{13}C labelled paraformaldehyde 23
5. One-pot-two-step conversion of CO into C_{5-6} carbohydrates....29
6. Figures of the electroreduction of CO into formaldehyde....31

1. Materials

1.1 Chemicals

3,4,5-trimethylthiazol-3-ium-2-ylidene **1**,[44] 1,3,4-triphenyl-4,5-dihydro-1*H*-1,2,4-triazol-l-ium-5-ylidene **2**[49] and 4,5-Dihydro-5-methoxy-1,3,4-triphenyl-1*H*-1,2,4-triazole **3**[49] were prepared according to literature procedures. Glycolaldehyde dimer, D,L-glyceraldehyde, dihydroxyacetone, D-erythrose, D-(-)-ribose, D-(+)-mannose, D-(-)-fructose, D-(+)-galactose, trimethylsilyl chloride, hydroxylamine hydrochloride, 1,4-butanediol, hexamethyldisilazane, potassium hydroxide, potassium chloride, potassium carbonate, potassium phosphate, pyridine, methanol and 37% aqueous solution of hydrogen chloride were purchased from commercial sources and used without further purification.

1.2 Procedures

Carbene **1** and **2** were handled following standard Schlenk line and glove box techniques using argon as the inert gas. Compound **3** was handled in Pyrex tubes with PBT screw caps under air atmosphere. Water was evaporated under reduced pressure (0.5 – 0.6 mbar) at room temperature using the Schlenk line until achieved constant pressures below 0.03 mbar. A homemade adaptor was used to connect Pyrex tubes to the Schlenk line. Deuterated solvents were flushed with Ar and stored under Ar over 4 Å molecular sieves. NMR spectra were collected on Bruker Avance III 400 HD. All chemical shifts for ^1H are relative to tetramethylsilane (TMS) and are given in ppm. Gas chromatography (GC) and mass spectrometry (MS) were performed with a Shimadzu GC-2010 plus GCMS-QP2010 with DI, using EI as ionization mode. The post-treatment was carried out with the open source OpenChrom software.[50] pH was measured with a Mettler Toledo pH-meter.

1.3 CoPc/MWCNT film preparation

1 mg/mL of multi-walled carbon nanotubes (MWCNTs, 6-9 nm diameter 5 µm length, > 95% Carbon, Sigma Aldrich) were dispersed in 10 mL ethanol (Analysis grade, Merck). This suspension was then used to suspend 0.1 mg/mL cobalt phthalocyanine (β form, 97% dye content, Sigma Aldrich), and sonication was performed for 30 min. Later, 10 µL of Nafion 5% solution (perfluorinated ion exchange Nafion© powder, 5 wt % solution in low aliphatic alcohol/H_2O, Sigma Aldrich) was added per mL of suspension followed by 30 min of sonication again. Then, the suspension was stirred overnight (16 h) before use. 100 µL/cm^2 (50 µL + 50 µL) of this suspension was drop-casted on the carbon paper (Freudenberg $H_{15}C_{13}$ – 22 x 30 cm, Lot # 4944-$H_{15}C_{13}$), and dried at room temperature overnight (16 h), yielding electrodes with 10 µg/cm^2 catalyst loading (17.5 nmol). The electrode was allowed to dry before adding a 50 µL second drop to prevent ink from spilling outside the active carbon paper surface (1x1 cm^2). The carbon paper was connected with a clip and protected with Teflon tape to leave a geometric film surface close to 1 cm^2. The drop casting was modified (seven times 50 µL) as required during optimization studies when 1.5 x 2 cm^2 carbon paper was used. It was kept at 11.66 µg/cm^2, leading to a catalyst loading of 20.4 nmol/cm^2. The exact geometric surface of the film was measured subsequently and used for current density calculation.

1.4 Electrolyte preparation

0.625 M potassium phosphate, monobasic (KH_2PO_4) was prepared by dissolving 1.361 g of KH_2PO_4 (MW = 136.1, Sigma Aldrich) in 16 mL of Milli-Q® water and 5 M potassium hydroxide (KOH, MW = 56.11, Emprove essential Merck) was prepared by dissolving 1.122 g of KOH in 4 mL of Milli-Q® water. Each pH solution was made prior to the experiment using prepared 0.625 M KH_2PO_4 and 5 M KOH for initial CV and controlled potential electrolysis studies. The exact pH was noted before the experiments using the Mettler Toledo FiveEasy pH meter FP20. In addition to it, for later required studies, 1.122 g KOH pellets were dissolved in 20 mL Milli-Q® water to prepare 1M KOH and 2.05 mL 37% HCl was added diluted with 2.95 mL Milli-Q® water to prepare 5 M HCl. KCl buffer was prepared using as prepared 20 mL 1 M KOH, which was diluted with 4 mL 5 M HCl. The desired solution (12.5 mL) was utilized from this KCl buffer, fresh every time, for which pH was set to 12 with utmost care.

1 M potassium oxalate solution was prepared by dissolving 369 mg of potassium oxalate monohydrate (MW = 184.23, Sigma Aldrich) in 2 mL of as prepared pH 12 phosphate or KCl buffer solution.

1.5 Stock solution for the formose reaction

Solution A: aqueous basic solution with pH was adjusted to 8, 12, 13 and 14 using a pH-meter utilizing a 5 M KOH solution and a 1 M HCl solution.

2. Methods

2.1 General procedure for reaction with commercial paraformaldehyde

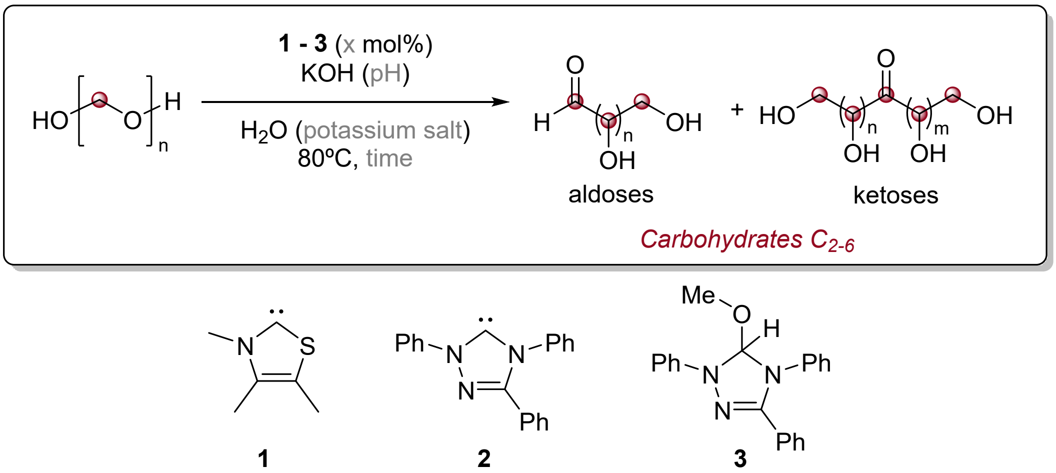

Scheme S1. Summary of the formose reaction using catalysts **1-3**.

Paraformaldehyde was placed in a Schlenk in an argon glovebox for **1** and **2** or in a Pyrex tube for **3** equipped with a magnetic stir bar and then charged with **1**, **2** or **3**. When the mass of catalyst (**1-3**) was equal or higher than 3 mg, the mass was measured directly with a balance. When the mass was smaller, a 1 mL stock solution of THF was prepared and the desired volume taken out with a micro-syringe, injected in the reaction vessel and the THF removed under reduced pressure. Solution A at pH 12 (Ar-flushed in case of **1** and **2**) was added to the reaction vessel. The solution was stirred at 80°C for the time of reaction and then cooled to room temperature.

Table S1. Preparation of the formaldehyde solution at various concentrations.

[HCHO] (mol/L)	m_{HCHO} (mg)	n_{HCHO} (mmol)	V_{water} (mL)
1	30	1	1
0.1	15	0.5	5
0.03	3	0.1	3.3
0.026	3	0.1	3.8
0.022	3	0.1	4.5
0.019	3	0.1	5.3
0.015	3	0.1	6.7
0.01	3	0.1	10

Table S1 contains the mass of paraformaldehyde and the volume of solution used to prepare the various concentrations. For the analysis of the formose reaction, the full sample was dried and further functionalized, at the exception of the 1 M and 0.1 M reactions for which aliquots of 0.1 and 1 mL,

respectively, were taken out for derivatization and GC/MS analysis (*vide infra*). The fact that only a part of the reaction was analyzed in these cases was taken into account in the yield calculation.

2.2 General method of controlled potential electrolysis

In the above-prepared electrolyte, the working electrode (WE) is set in a three-electrode configuration with a saturated calomel electrode (SCE, Hach) as the reference electrode (RE) and a platinum mesh (Goodfellow) as the counter electrode (CE). The latter was positioned inside an anolyte compartment bridge (containing the same electrolyte with 1 M Potassium Oxalate), separated from the catholyte by a glass frit. Applied potential conversion from SCE to RHE was calculated following E (V *vs.* RHE) = E (V *vs.* SCE) + 0.244 + 0.059 pH. Gas of interest, either Ar or ^{12}CO (Air liquid) was flushed for 20 min before starting electrolysis *via* needles through a rubber septum. After a first cyclic voltammetry (CV) scan performed from 1 to -1 (V vs. RHE) at 100 mV/s to check the system, controlled potential electrolysis (CPE) experiment was performed using a VIONIC potentiostat (Metrohm).

2.3 Final optimized procedure to electrochemically accumulate higher amounts of HCHO

Two 1.5 x 3 cm^2 carbon electrodes were prepared. A 1.5 x 2 cm^2 active area was loaded with CoPc catalytic ink (7 times 50 µL) and dried overnight at room temperature. The electrolyte solution was made by mixing final volumes, 20 mM 1 M KOH (prepared dissolving 1.234 g in 22 mL water, pH = 14) and added with stepwise 5 M HCl (prepared using 2.07 mL of 12.1 M HCl added to 2.93 mL water) with exact volumes and recorded pH in bracket, to reach pH 12, 1 mL (13.84) + 1 mL (13.51) + 0.5 mL (13.24) + 200 µL (13) + 200 µL (12.64) + 100 µL (12.25) + 50 µL (11.77), later balanced again with 1 M KOH with volumes and pH, 40 µL (11.84) + 40 µL (11.92) + 10 µL (11.94) + 10 µL (11.96), this gives a final volume as 20.1 mL 1 M KOH and 3 mL 50 µL 5 M HCl to reach the desired pH of 12. The solution was stored overnight. The next day, the pH was measured again and brought back to exact pH 12 by adding 26 µL 5M HCl and 10 µL 1 M KOH. The as-prepared electrolyte (2 mL) containing 1 M potassium oxalate (368.5 mg) was used in the counter electrode chamber, 2.5 mL of electrolyte in the reference electrode, and 12.5 mL of electrolyte in the working electrode chamber. Argon was bubbled for 15 min, and then CO will be bubbled for 20 min. CV was recorded to see the characteristic CO activation CV wave. Controlled potential electrolysis (CPE1) was carried out for 7 h.

After CPE1 the pH reached 13.24 and the solution volume was 11.2 mL. A 392 µL sample was collected for NMR analysis, possibly splitted in two for comparative analysis, and later 140 µL 5M HCl and 93 µL 1 M KOH was added to reach pH 11.96. The solution was transferred back to the cell, and pH was remeasured as 12.01. The electrode was replaced with a fresh one, followed by 15 min argon and then 20 min CO bubbling, and CPE2 was carried out for extra 8 h.

The same exact procedure was repeated for the consecutive CPE3 (8 h) and CPE4 (8 h).

After CPE4, pH reached 13.85 and the solution volume was 6.2 mL. A 196 µL sample was collected for NMR analysis and later 270 µL 5M HCl and 20 µL 1M KOH was added to reach pH 12.03.

2.4 Method of formaldehyde and methanol quantification

After CPE, 500 µL of the electrolyte was mixed with an equivalent volume of 1 M $NaHSO_3$ (ACS reagent, Sigma Aldrich) and stirred to form the HCHO-bisulphite adduct. Out of this mixture, 392 µL were taken and mixed with 48 µL of D_2O (99% atoms D, Sigma Aldrich) and 40 µL of 4mM $DMSO_2$ in an NMR tube. A minimum of 64 ^1H-NMR scans were accumulated (Bruker 400 MHz NMR or Bruker B-ACS 60 Ultrashield 300 MHz NMR) with water pre-saturation (zgpr) method and using a relaxation time of 2 s. The product amount is measured with respect to the internal standard $DMSO_2$.

2.5 Method of characterization of carbohydrates

2.5.1 Derivatization and GC/MS analysis

The mixture of carbohydrates and polyoxygenated compounds produced by the formose reaction was analyzed by GC-MS method, requiring a derivatization step to afford soluble and volatile product to analyze (**Figure S1**).[51-57] This derivatization step consists i) in the oximation of the carbonyl groups using hydroxylamine and ii) the silylation of the hydroxyl groups using hexamethyldisilazane (HMDS) and trimethylsilyl chloride (TMSCl). It has to be noted that the oximation step produces Z/E oxime mixture exhibiting close but different retention time in GC (see for example **Figure S2** with commercial carbohydrates).

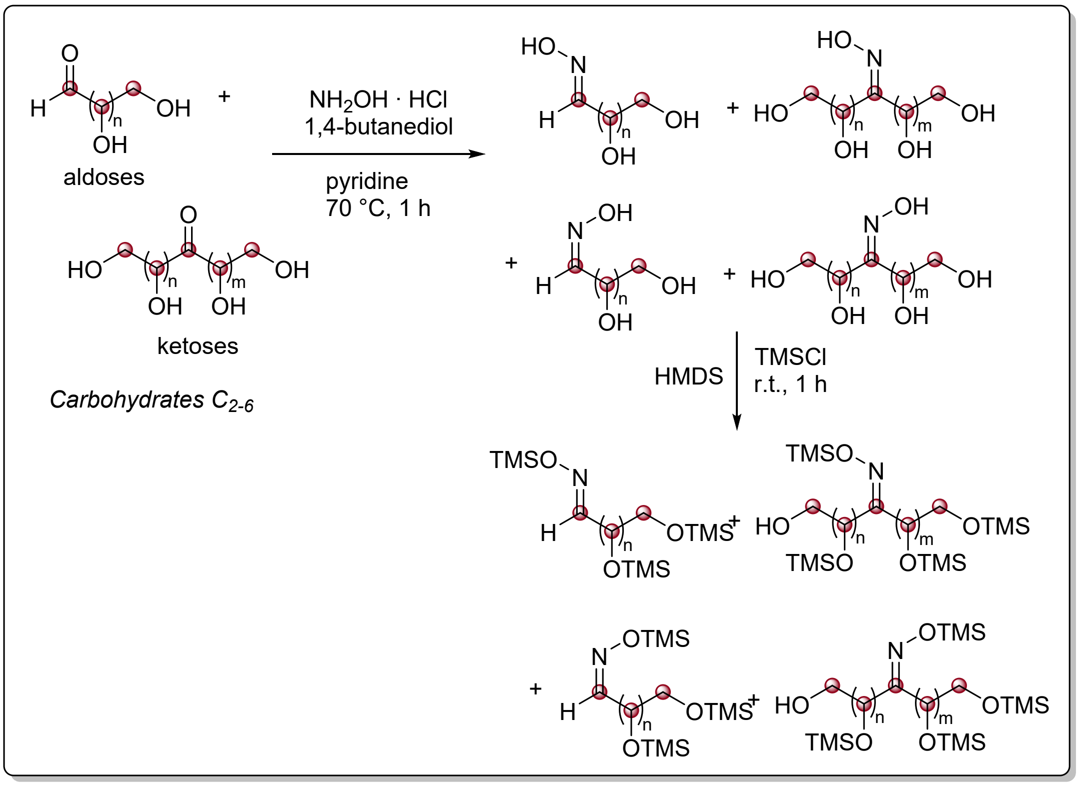

Figure S1. Derivatization of aldoses and ketoses.

The reaction mixture obtained after the formose reaction was completely dried by evaporating the water. A solution of oxime containing 1,4-butanediol as internal GC standard was prepared according to literature[44] and added (0.4 - 1.3 mL) to the reaction which was stirred at 70°C for 1 h (**Figure S1**). A fresh oxime solution was prepared every 3-4 weeks. After cooling to room temperature, HMDS (420 µL) and TMSCl (255 µL) were added. The reaction was stirred 1 h at room temperature, resulting in a solution with a white precipitate. Dichloromethane (6 mL) were added to dilute the sample and an aliquot of 1.5 mL was filtered over a 0.45 µm pore size PTFE syringe filter. The aliquot was then analyzed by GC-MS (1 µL sample, 30 m ZB-5M plus column, temperature program: 1 min at 50°C, single ramp from 50°C to 250°C with a heating rate of 2°C/min, and then 19 min at 250°C).

We analyzed the commercially purchased glycolaldehyde dimer, D,L-glyceraldehyde, dihydroxyacetone (DHA), D-erythrose, D-(-)-ribose, D-(+)-mannose, D-(-)-fructose and D-(+)-galactose by GC/MS after derivatization. **Figure S2** shows the obtained chromatograms indicating distinct area for each carbon-chain length. **Figure S3** shows the MS spectrum of D-(-)-ribose. This spectrum was chosen to describe the fragmentation of an aldopentose. The peaks at m/z of 525, 510 and 420 are attributed to the molecular peak M^+, $[M-CH_3]^+$ and $[M-CH_3-HOTMS]^+$ respectively.[58] Smaller fragments come from homolytic

cleavages between two *sp*^{3} carbons[56,59] and the peaks at m/z of 147 and 73 are common peaks associated to the silylation step.[57,60]

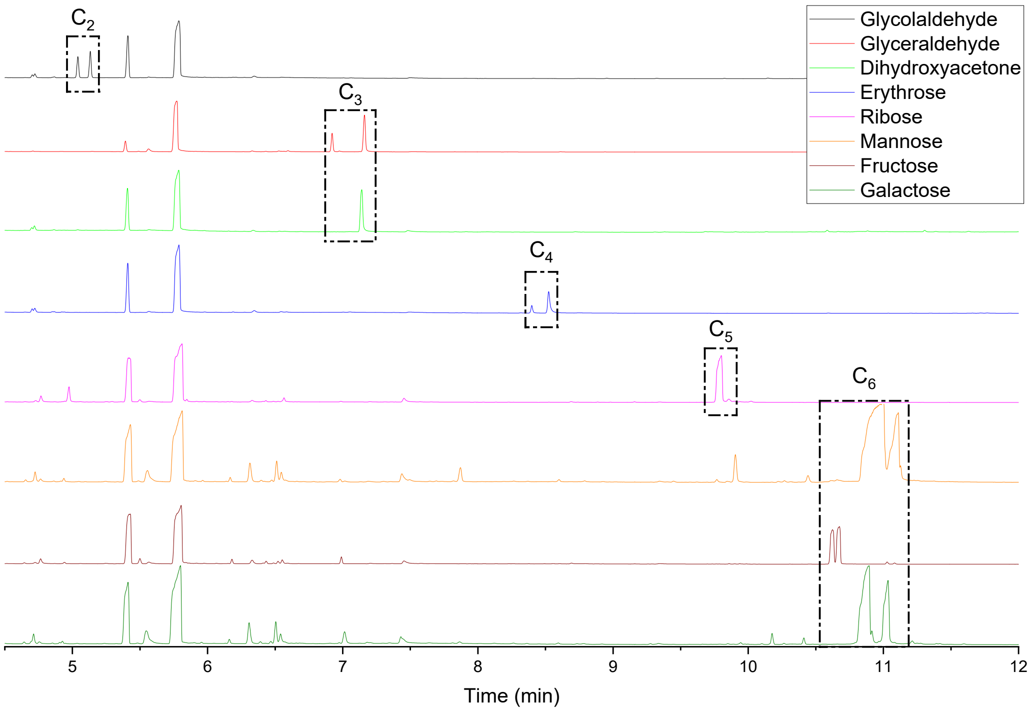

Figure S2. Chromatograms of commercial carbohydrates (hydroxylamine at 5.41 min and 1,4-butanediol at 5.78 min)

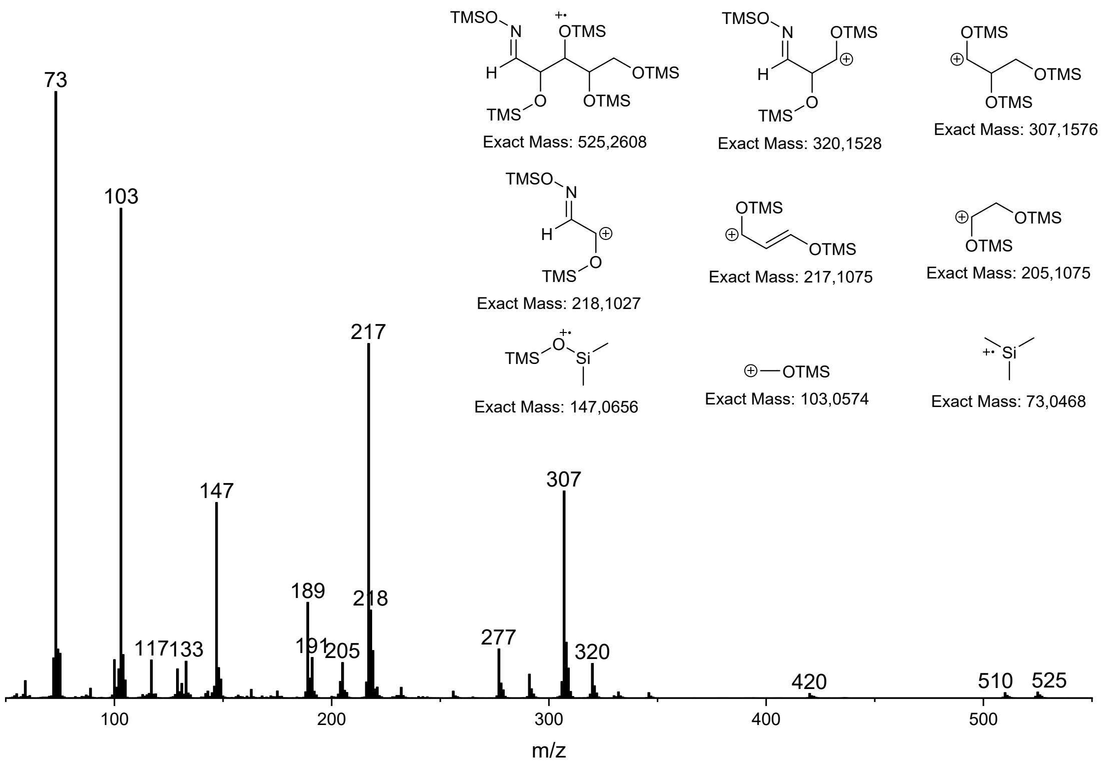

Figure S3. MS spectrum and principal fragmentations of commercial D-(-)-ribose after derivatization.

In the reaction mixture, the attribution of the peaks to carbohydrate was made based on the retention time and the MS fragmentation reported in the literature (**Table S2**).[53] Aldoses and ketoses can be differentiated because the cleavage of the bond adjacent to the oxime function is less favorable than the other C-C bonds. As a consequence, small fragments corresponding to terminal oxime will be observed in case of aldoses.[61]

Table S2. Main fragments of oximated and trimethylsilylated C_{2}-$_{6}$ carbohydrates

Compound	Molecular peak	Reported m/z fragments[6]
C_2	219	204; 130
C_3 Aldose	321	321; 306; 218; 191
C_3 Ketose	321	321; 306
C_4 Aldose	423	423; 408; 320; 219; 205
C_4 Ketose	423	423; 408; 320; 231; 191
C_5 Aldose	525	525; 510; 320; 307; 217
C_5 Ketose	525	525; 510; 422; 321; 231; 205
C_6 Aldose	627	612; 319; 217; 205; 157; 129
C_6 Ketose	627	621; 422; 307; 217; 103
C_7 Aldose	729	714; 422; 331; 217; 191
C_6 Alditol	614	614; 319; 217; 129
C_6 Aldonic Acid	628	613; 435; 359; 333; 319; 305; 292; 217

2.5.2 Calibration curves

Calibration curves were made for the quantification of carbohydrates with glycolaldehyde dimer, D,L-glyceraldehyde, DHA, D-erythrose, D-(-)-ribose, D-(-)-fructose and D-(+)-galactose. D-erythrose, D-(-)-ribose, D-(-)-fructose and D-(+)-galactose were chosen as representative carbohydrates for the $C_{4\text{-}6}$ because of their availability. Moreover, we confirmed that the calibration curves of the D-(-)-fructose and D-(+)-galactose showed negligeable differences for the slope and the y-intercept. Due to solubility issue, D-(+)-mannose did not afford clean curves. New calibration curves were produced every three months without significant variations. One example for each carbohydrate is shown in **Figure S4** to **Figure S9** (IS: internal standard).

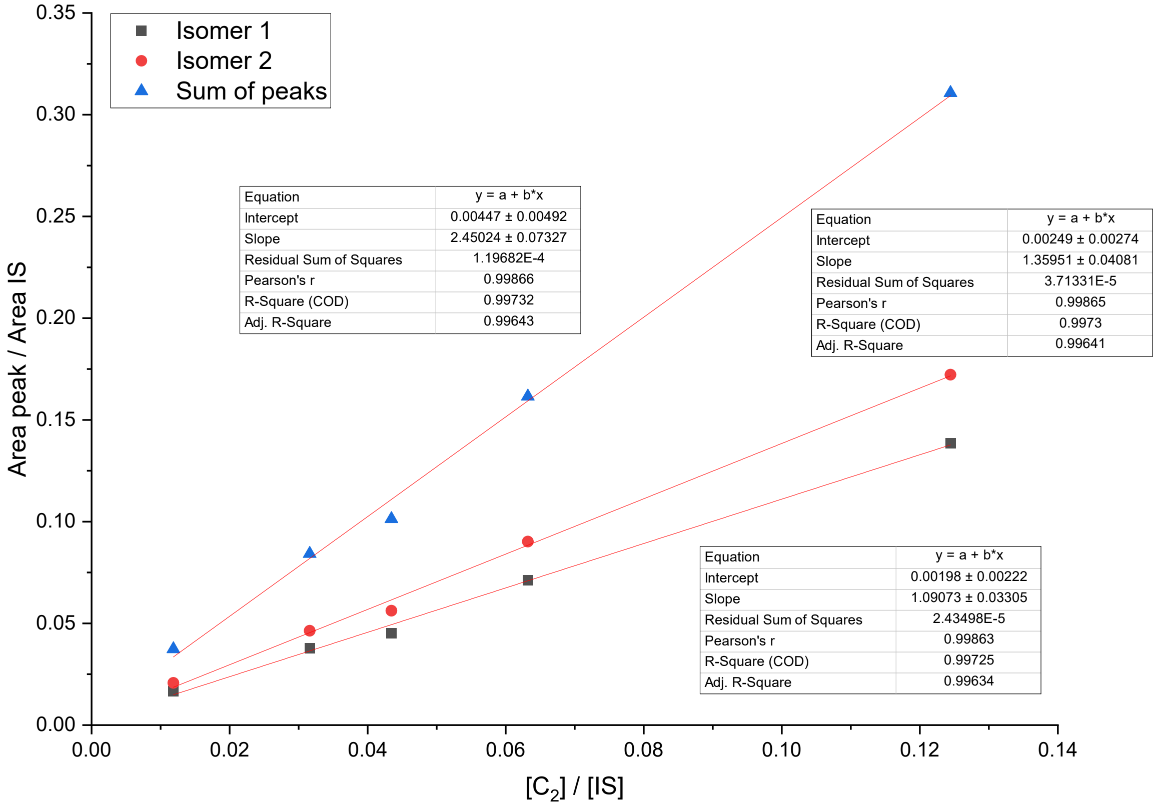

Figure S4. Calibration curve for glycolaldehyde.

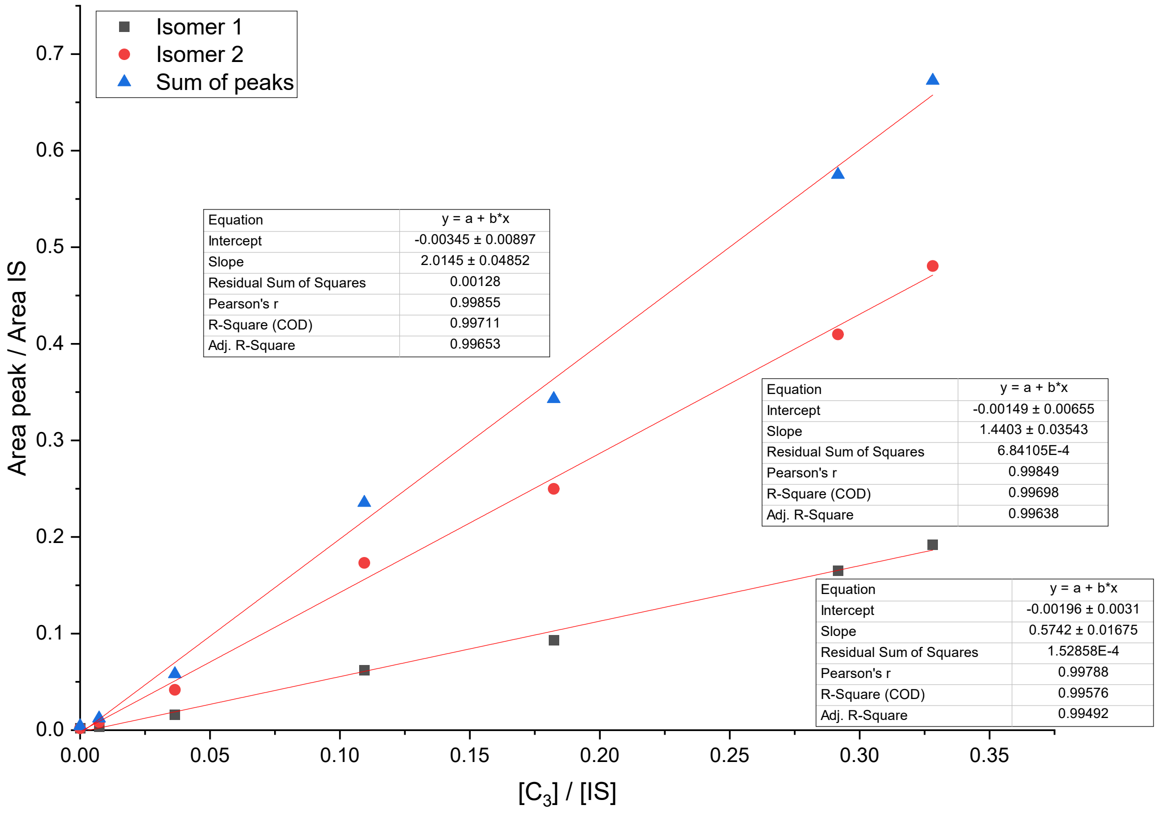

Figures S5. Calibration curve for glyceraldehyde.

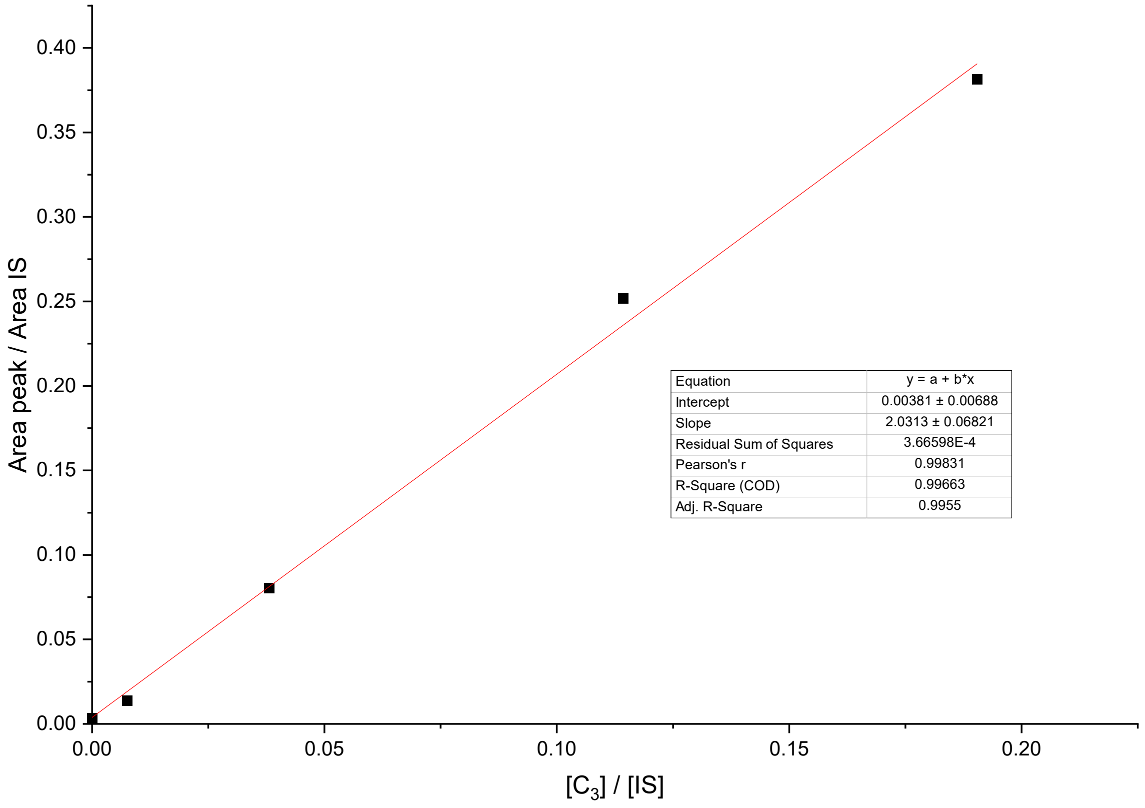

Figure S6. Calibration curve for DHA.

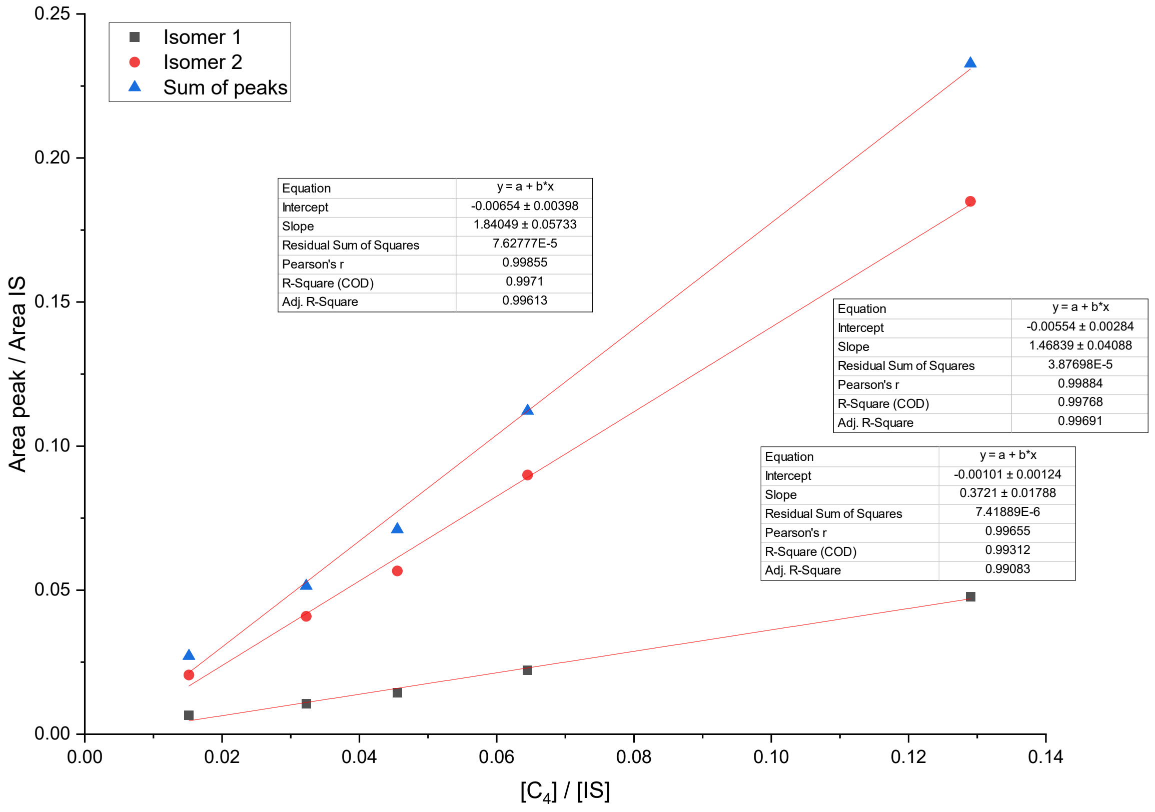

Figure S7. Calibration curve for erythrose.

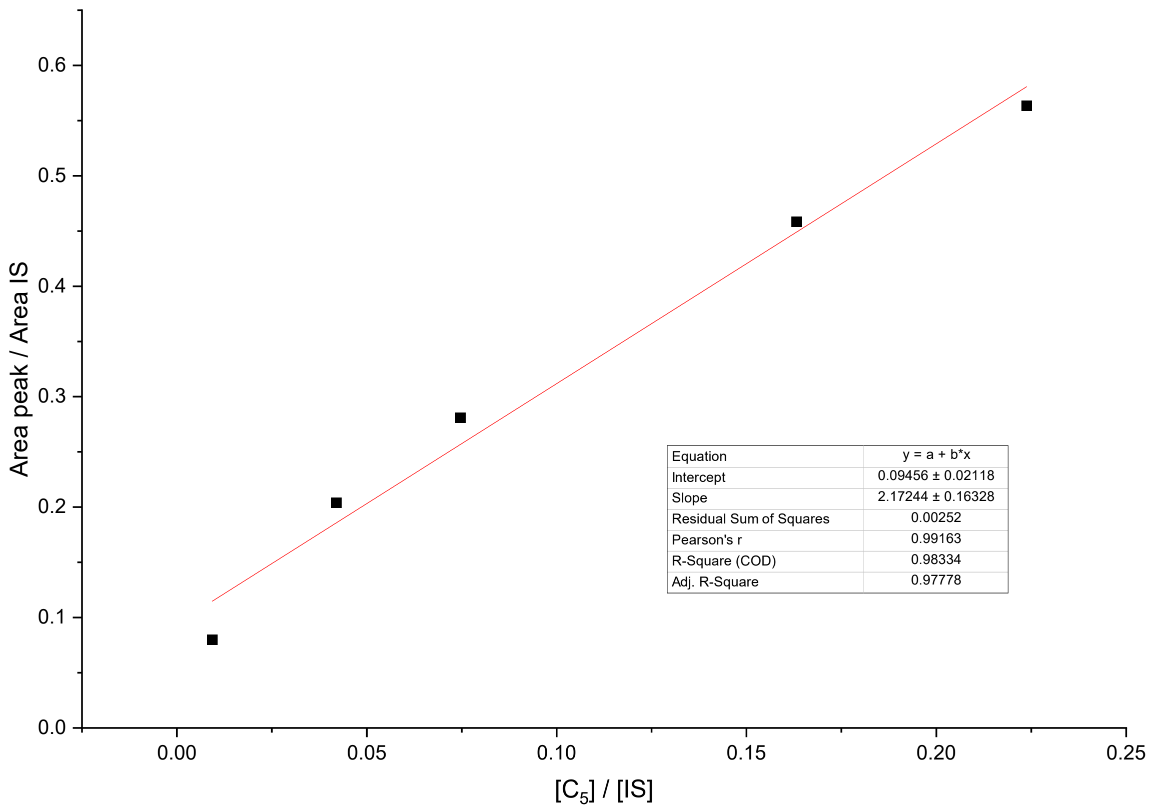

Figure S8. Calibration curve for ribose.

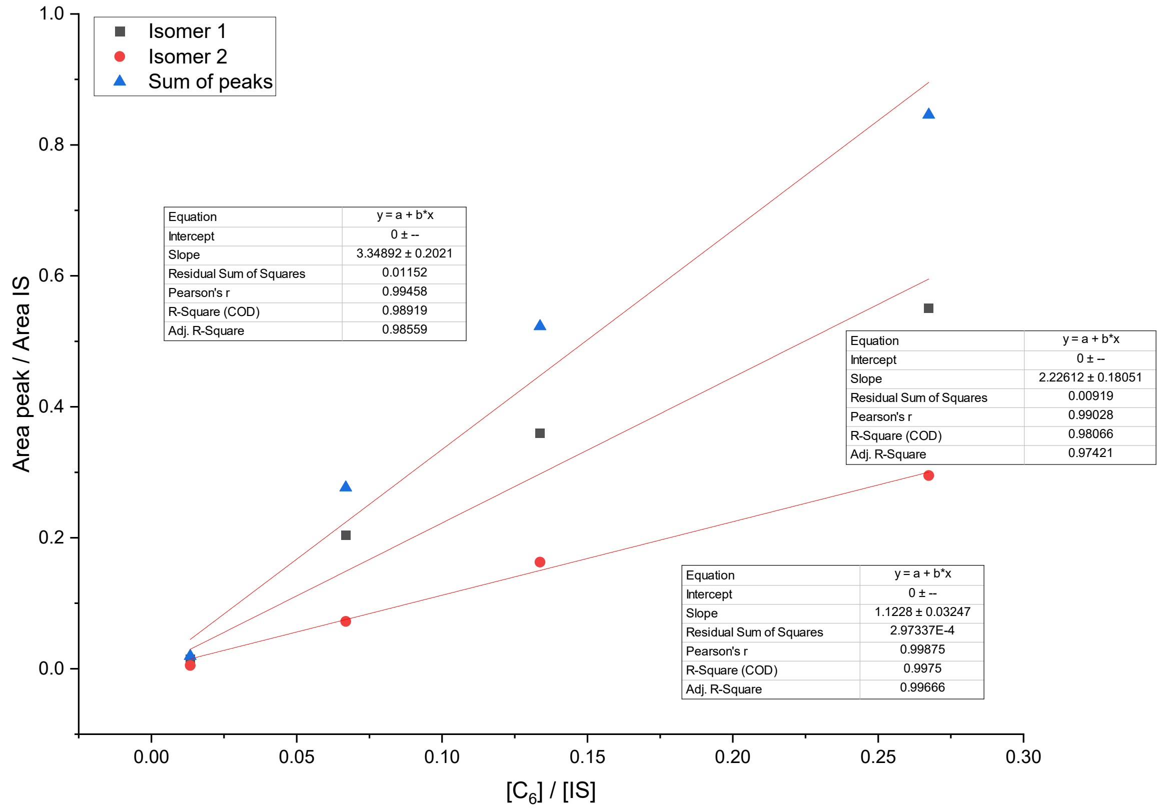

Figure S9. Calibration curve for galactose.

2.5.3 Yield calculation

Yields are then calculated against 1,4-butanediol. This procedure is a modification of that developed by Anderle.[57] For linear regimes, the equation of these curves is given by :

$$\frac{A_{Ck}}{A_{IS}} = a * \frac{[C_k]}{[IS]} + b \Leftrightarrow [C_k] = \frac{\left(\frac{A_{Ck}}{A_{IS}} - b\right) * [IS]}{a}$$

where A_{Ck} and A_{IS} are the areas under the GC peak of the carbohydrate C_k (k being the carbon chain length) and the 1,4-butanediol respectively, a and b are the slope and the y-intercept respectively of the calibration curves. [IS] is the concentration of the internal standard in the GC vial.

Carbohydrates C_k yields were calculated using the following equation:

$$Yield = \frac{\left(\frac{A_{Ck}}{A_{IS}} - b\right) * [IS] * V_{GC} * k}{a * n_{deriv}}$$

where V_{GC} is the total volume in the GC vial and n_{deriv} is the theoretical number of moles of formaldehyde transformed and derivatized. As [IS] also depends of V_{GC}, the final equation used is:

$$Yield = \frac{\left(\frac{A_{Ck}}{A_{IS}} - b\right) * n_{IS} * k}{a * n_{deriv}}$$

where n_{IS} is the number of moles of internal standard in the mixture.

2.5.4 Detailed calculation on one example

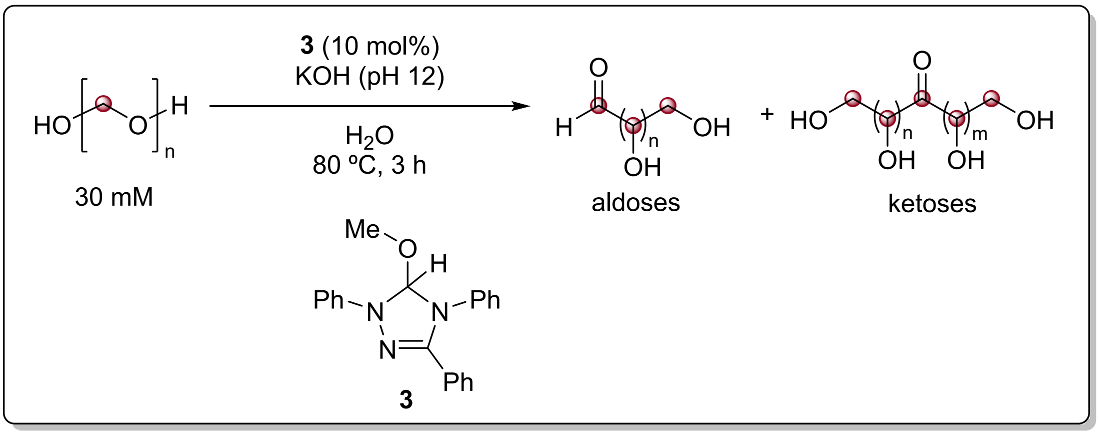

Scheme S2. Formose reaction with carbene catalyst **3** at 10 mol%.

The formose reaction using 30 mM of formaldehyde and 10 mol% of **3** and performed at 80°C for 3 h (**Scheme S2**) was chosen as an example to detail the complete characterization and yield calculation. Detailed calculation will be given for C_5 carbohydrates. The GC chromatogram of this reaction is shown in **Figure S10**. In the grey box of **Figure S10**, we have selected peaks with retention times between 9.45 min and 9.73 min which presumably belong to C_5 chains based on the retention time. Commercial D-(-)-Ribose exhibits a retention time of 9.79 min using the same GC-MS equipment and MS spectrum as shown in **Figure S2**. Since D-(-)-Ribose is the only commercial C_5 carbohydrate that we analyzed, the retention time is not sufficient to determine which other retention times belongs to the other C_5

carbohydrates, although they will exhibit similar retention times. MS analysis is thus used to identify all the GC peaks corresponding to C_5 carbohydrates.

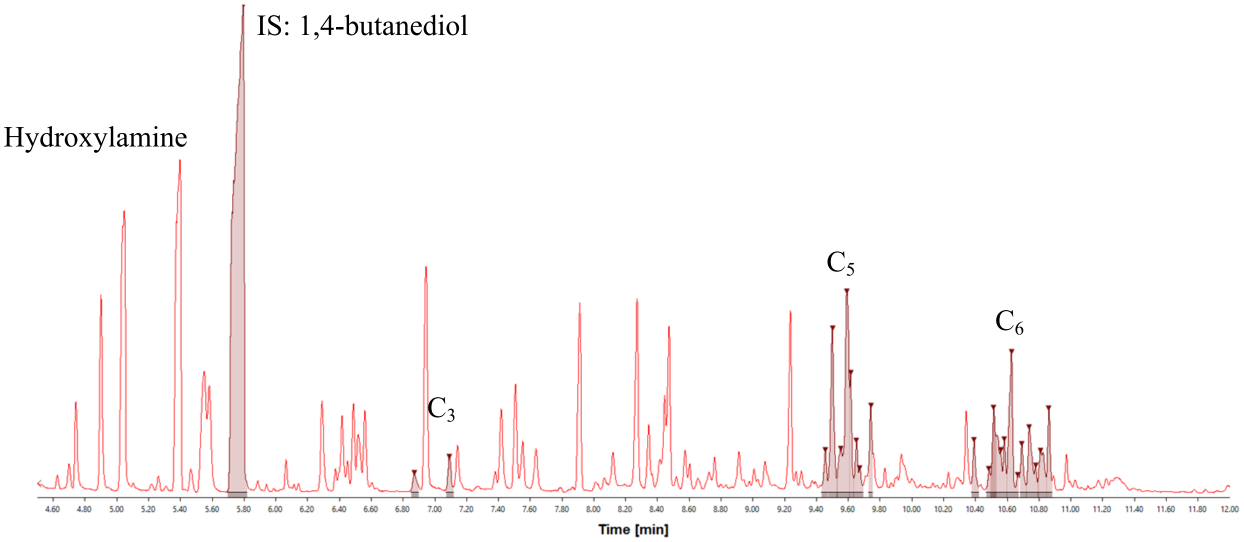

	RT [min]	Area	Start RT [min]	Stop RT [min]	Area [%]
**I.S**	5.795	2570507064	5.693	5.828	48.366
**C_3**	6.872	43956637	6.845	6.905	0.827
	7.092	61395242	7.062	7.115	1.155
**C_5**	9.457	69371510	9.428	9.473	1.305
	9.5	303768104	9.473	9.527	5.716
	9.553	100246808	9.527	9.567	1.886
	9.592	432395362	9.565	9.612	8.136
	9.615	154018206	9.607	9.633	2.898
	9.652	72219450	9.633	9.663	1.359
	9.673	32987505	9.667	9.695	0.621
	9.742	128531423	9.72	9.755	2.418
**C_6**	10.392	92111897	10.37	10.413	1.733
	10.485	38630057	10.467	10.497	0.727
	10.515	231815099	10.497	10.55	4.362
	10.558	58619333	10.55	10.57	1.103
	10.582	87492842	10.565	10.597	1.646
	10.627	289531190	10.593	10.65	5.448
	10.692	95530916	10.673	10.722	1.797
	10.738	152222035	10.717	10.77	2.864
	10.778	43601692	10.768	10.795	0.82
	10.81	66843162	10.792	10.818	1.258
	10.825	64506360	10.818	10.843	1.214
	10.862	124366439	10.847	10.883	2.34

Figure S10. GC chromatogram with the peak report of the formose reaction using 30 mM of formaldehyde, 10 mol% of **3** at 80°C for 3 hours.

In the present example, the MS spectra of the peaks between 9.45 min and 9.73 (**Figure S11**) were analyzed and compared to the MS spectrum of the ribose. The fragments identified for the commercial ribose are present in the MS spectra associated with the peaks having a retention time of 9.48, 9.50,

9.59, 9.62, 9.65 and 9.74 min. These compounds are thus identified as aldopentoses. The compounds with retention times of 9.55 and 9.67 min have not the exact same fragmentation patterns than an aldopentose. In the work of Trapp *et al.*,[53] aldoses were distinguished from ketoses and the main fragments reported in **Table S2**. This differentiation is possible thanks to the oximation step because a cleavage of the adjacent bond to the oxime function is less favorable, leading to different fragmentation with aldose or ketose.[61] Fragments associated with the C_5 ketose are present in the MS spectra of the compounds at 9.55 and 9.67 min. These two compounds are thus identified as ketopentoses.

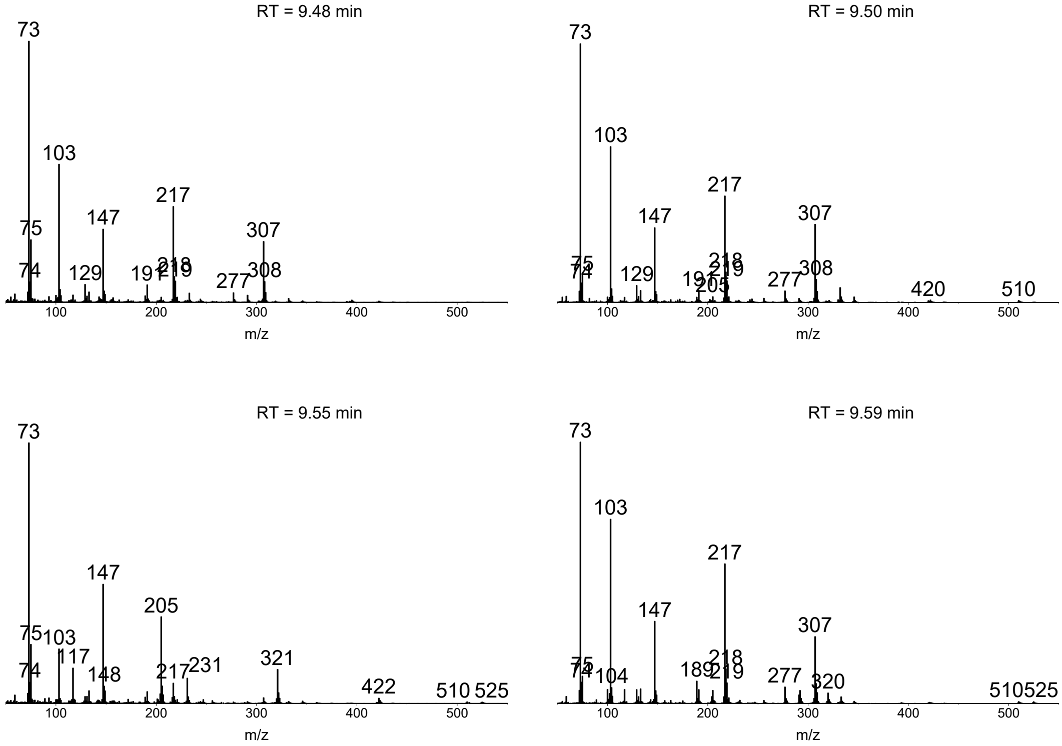

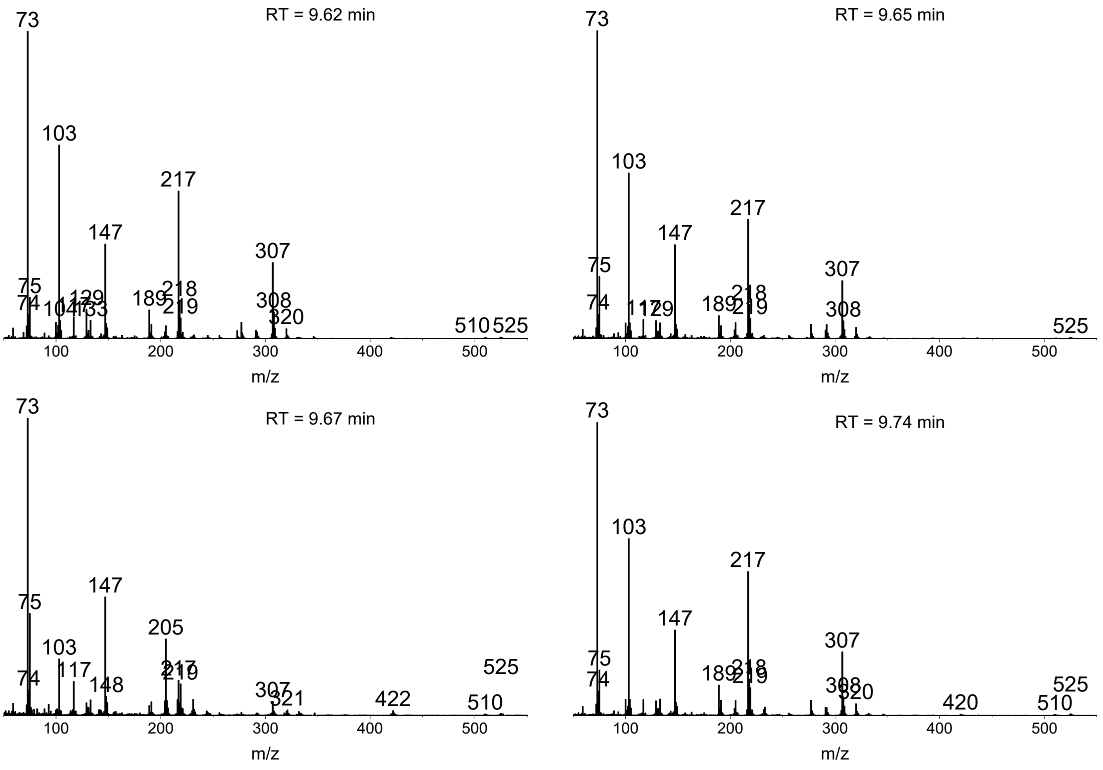

Figure S11. MS spectra of the C_5 peaks in the chromatogram of the formose reaction relative to the chromatogram of **Figure S10**.

The sum of the peaks area for the C_5 aldopentose and ketopentoses led to a total yield of 31% taken into account 0.6 mL of the oxime solution, containing 125 mg of 1,4-butanediol).

Table S3. Sum of the areas measured of the internal standard (IS) and carbohydrates peaks with OpenChrom.

A_{IS}	A_{C2}	A_{C3}	A_{C4}	A_{C5}	A_{C6}
2570507064	0	105351879	0	1293538368	1345271022

$$Yield = \frac{\left(\frac{1293538368}{2570507064} - 0.0946\right) * 0.0332 * 5}{2.1724 * 0.1} = 0.31$$

The same procedure was applied with the carbohydrates having different carbon-chain lengths.

3. Formose reaction with commercial paraformaldehyde

3.1 Initial evaluation of the formose reaction catalyzed by 1-3 at 1, 0.1 and 0.01 M HCHO

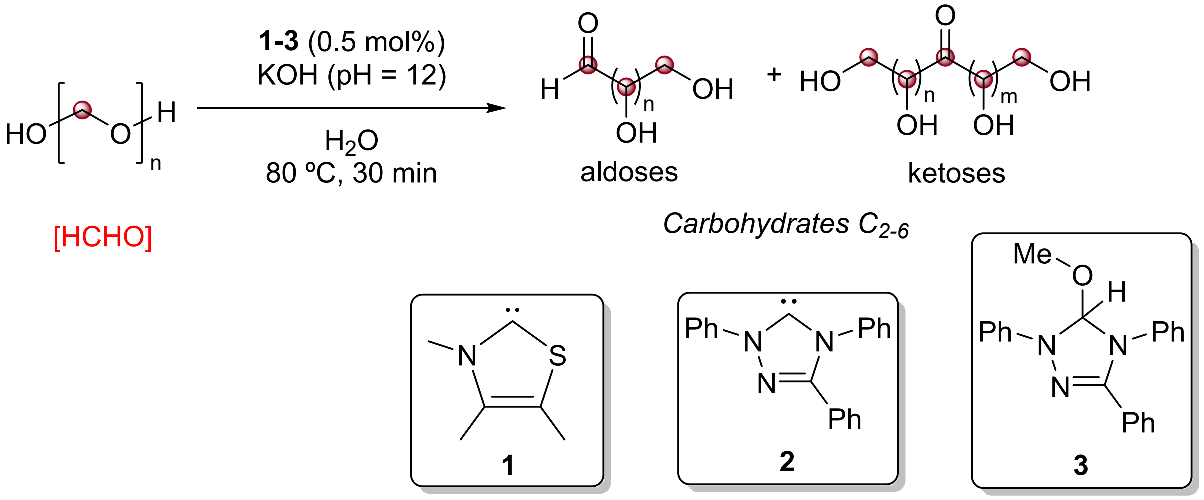

Scheme S3. Formose reaction with catalysts **1-3** (0.5 mol%) at different concentrations of formaldehyde.

Following the general procedure, initial evaluations with 0.5 mol% of **2** and **3** were conducted within 30 min at 80°C, pH 12 at [HCHO] = 1, 0.1 and 0.01 M (**Scheme S3**). **Figures S12-13** show the stacked chromatogram obtained with **2** or **3** as catalyst. **Table S4** gathers the calculated yields.

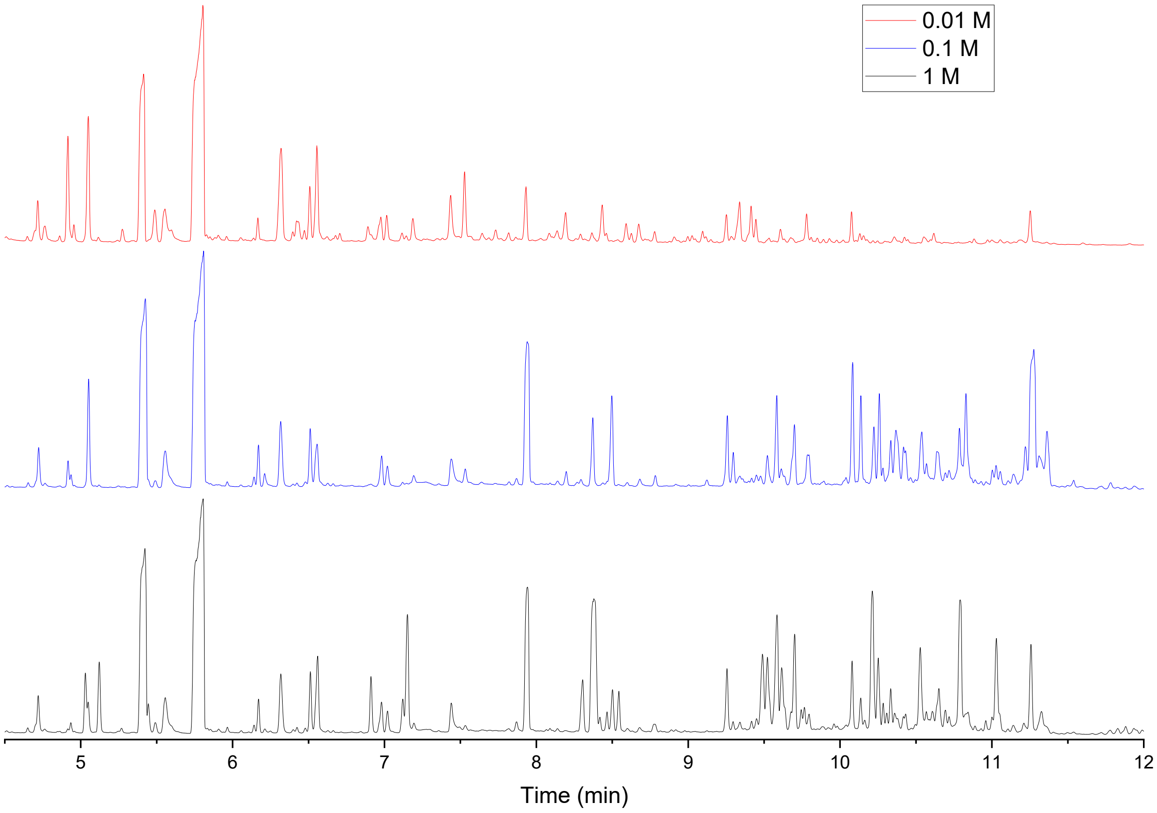

Figure S12. Chromatograms of formose reactions using **2** at 0.01, 0.1 and 1 M of formaldehyde.

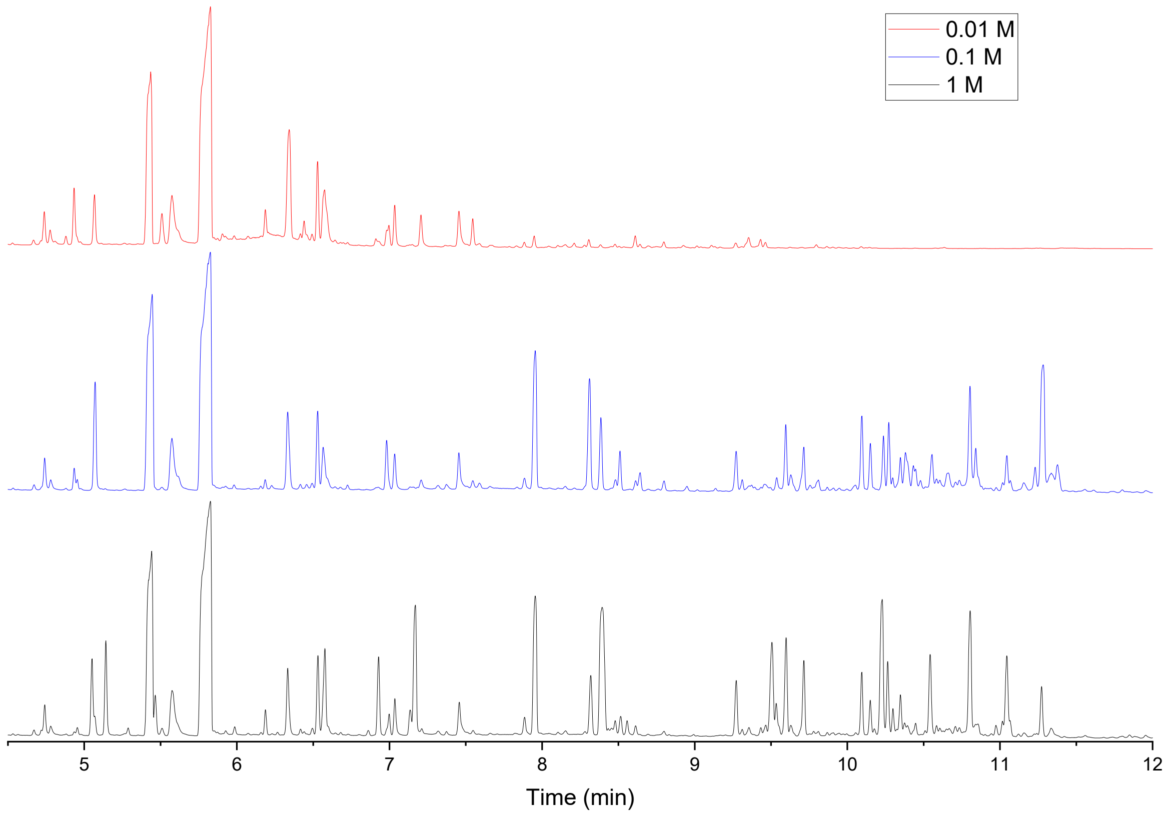

Figure S13. Chromatograms of formose reactions using **3** at 0.01, 0.1 and 1 M of formaldehyde.

Table S4. Carbohydrate yields of the formose reaction using none, **1**, **2** or **3** as catalyst.

[HCHO] (mol/L)	Catalyst	C_2 %	C_3 %	C_4 %	C_5 %	C_6 %	Total %
**1**	**1**	0	0	0	0	0	0
	**2**	2	6	27	34	13	82
	**3**	4	8	29	22	8	71
	-	0	0	0	0	0	0
**0.1**	**1**	0	0	0	0	0	0
	**2**	0	0	5	9	17	31
	**3**	0	0	5	6	15	26
	-	0	0	0	0	0	0
**0.01**	**2**	0	< 1	< 1	0	0	< 1
	**3**	0	0	1	0	0	1
	-	0	0	0	0	0	0

3.2 Influence of the pH

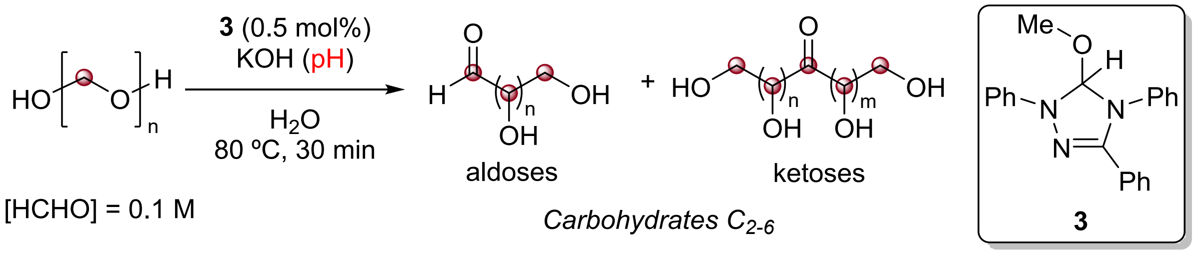

Scheme S4. Formose reaction with catalysts **3** (0.5 mol%) at different pH.

Following the general procedure, evaluation of the pH was conducted with 0.5 mol% of catalyst **3** within 30 min at 80°C, at 0.1 M of formaldehyde (**Scheme S4**). Table S5 indicates the calculated yields.

Table S5. Chemical yields of the formose reaction at different pH values.

**pH**	**C_2 %**	**C_3 %**	**C_4 %**	**C_5 %**	**C_6 %**	**Total %**
14	0	1	2	2	0	5
13	0	< 1	0	0	0	< 1
12	0	0	5	6	15	26
8	< 1	1	10	3	5	19

3.3 Effect of the potassium cation

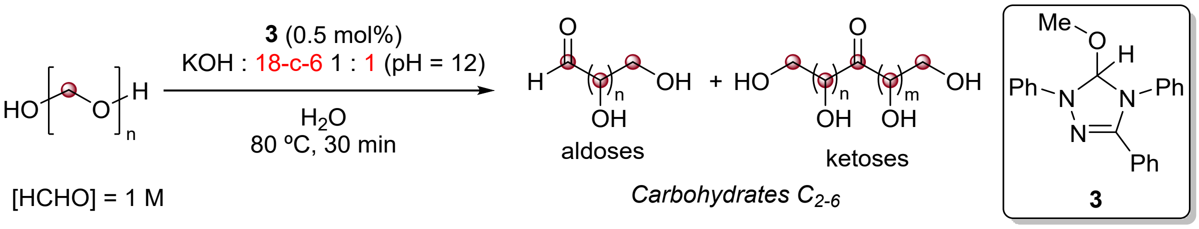

Scheme S4. Formose reaction with catalysts **3** (0.5 mol%) with crown ether 18-c-6.

Alkali cations are known to participate in aldol and retro-aldol reactions which might be at play in such transformation and impact the selectivity.[46,47] When the reaction was carried out at [HCHO] = 1 M with and without one equivalent of 18-c-6 related to KOH (**Scheme S4**), no difference was observed (**Figure S14**). This feature is in line with the fact that K^+ is one of the least impactful cations in the reaction as compared to Ca^{2+} for instance.[37]

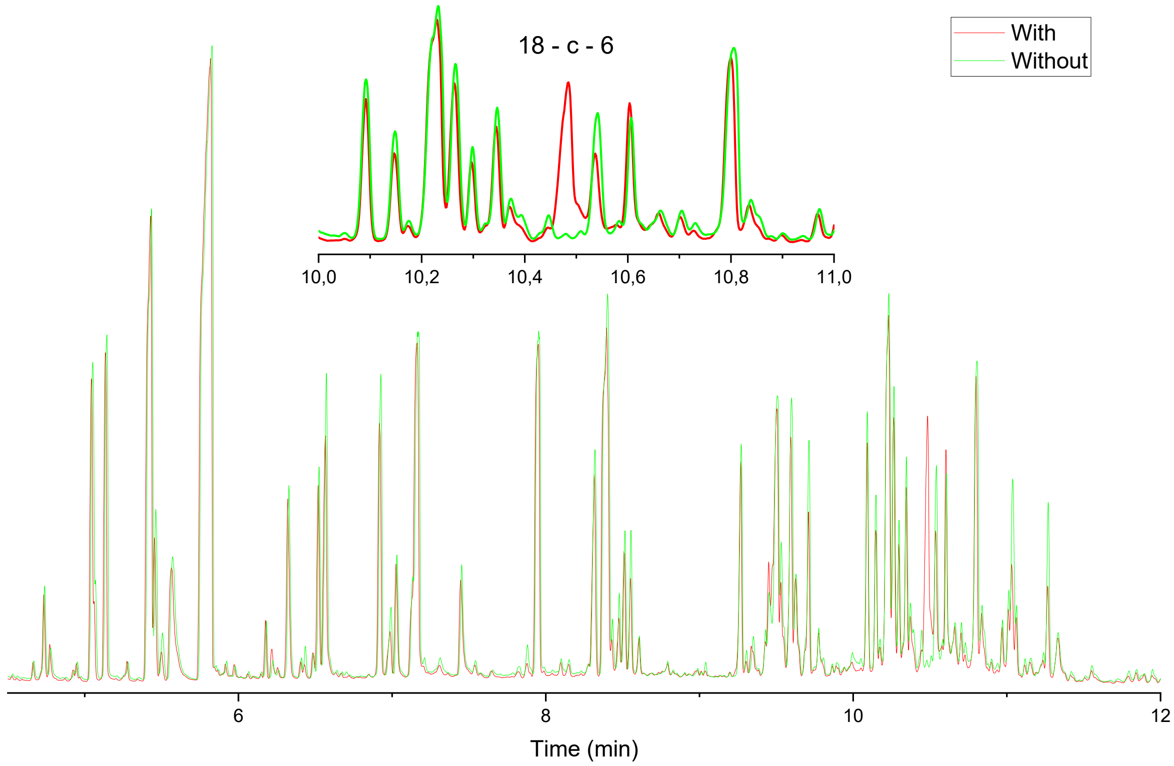

Figure S14. Chromatograms of formose reactions conducted with 18-crown-6 (red) and without 18-crown-6 (green).

3.4 HCHO concentration, reaction time and catalyst loading

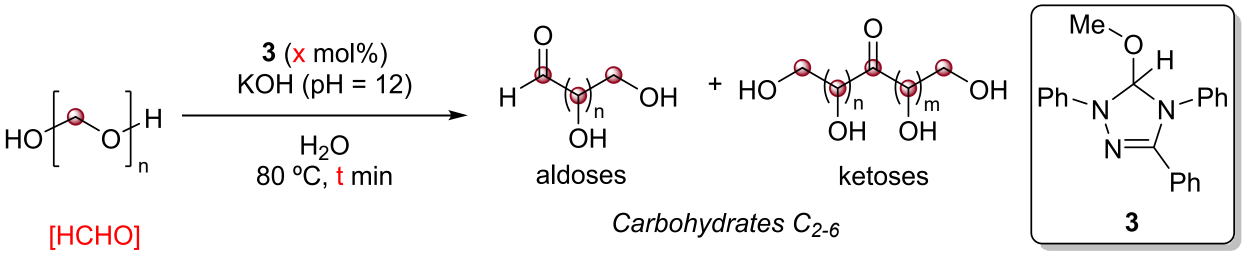

Scheme S5. Optimized formose reaction with catalysts **3**.

After the initial evaluation of the formose reaction, a more complete optimization study was conducted with catalyst **3** at pH 12 and 80°C (**Scheme S5**). The time of reaction, catalyst loading and formaldehyde concentration as well as the reproducibility were evaluated. The results are indicated in **Table S6**.

Table S6. Chemical yields of the formose reaction screening the concentration of formaldehyde, reaction time and catalyst loading using **3** (* using **1** as catalyst).

Entry	[HCHO] (mM)	Time (min)	Cat. loading (mol%)	C_2 %	C_3 %	C_4 %	C_5 %	C_6 %	Total %
**1**	10	30	0.5	0	< 1	0	0	0	< 1
**2**	10	180	0.5	0	< 1	0	0	0	< 1
**3**	10	30	5	0	< 1	2	< 1	0	2
**4**	10	180	5	0	< 1	0	0	0	< 1
**5**	10	30	10	0	1	1	0	0	2
**6**	10	180	10	0	0	0	0	0	0
**7**	10	30	20	0	1	1	0	0	2
**8**	10	180	20	0	0	1	0	0	1
**9**	30	30	0.5	0	< 1	0	0	0	< 1
**10**	30	90	0.5	0	< 1	0	< 1	12	12
**11**	30	180	0.5	0	< 1	0	5	12	18
**12**	30	30	10*	0	0	0	0	0	0
**13**	30	90	10	0	0	0	21	20	41
**14**	15	90	10	0	0	0	< 1	2	2
**15**	19	90	10	0	0	0	9	9	18
**16**	22	90	10	0	0	0	9	9	18
**17**	26	90	10	0	0	0	12	9	21
**18**	30	90	10	0	0	0	27	26	53
**19**				0	0	0	19	16	35
**20**				0	0	6	26	14	46
**21**				0	0	6	29	15	50
**22**				0	0	0	29	22	51
**23**				0	0	0	21	20	41
**24**				0	0	0	17	13	30
**25**				0	0	0	18	18	36
**26**	100	30	0.5	0	0	5	6	15	26
**27**	100	30	10	0	3	15	43	23	84

3.5 HCHO consumption

The crude mixture of the formose reaction at 15 mM **Figure S15** and 22 mM **Figure S16** were analyzed by ^{1}H NMR in order to quantify the remaining amount of formaldehyde and determine if formaldehyde was fully converted or not to explain the low yields. For the experiment at 15 mM, aliquots were collected after 45 min, 90 min and 7 h of reaction and at 22 mM, aliquots were collected after 90 min and 7 h. Following the procedure reported in the literature, sodium hydroxy methane sulfonate was added to convert formaldehyde into a bisulfite adduct featuring a 1H NMR chemical shift at 4.40 ppm.[35] As shown in **Figure S15** and **Figure S16**, only a small amount of formaldehyde was observed after 45 min for the reaction with 15 mM, but not in the other cases, indicating that formaldehyde has been fully converted.

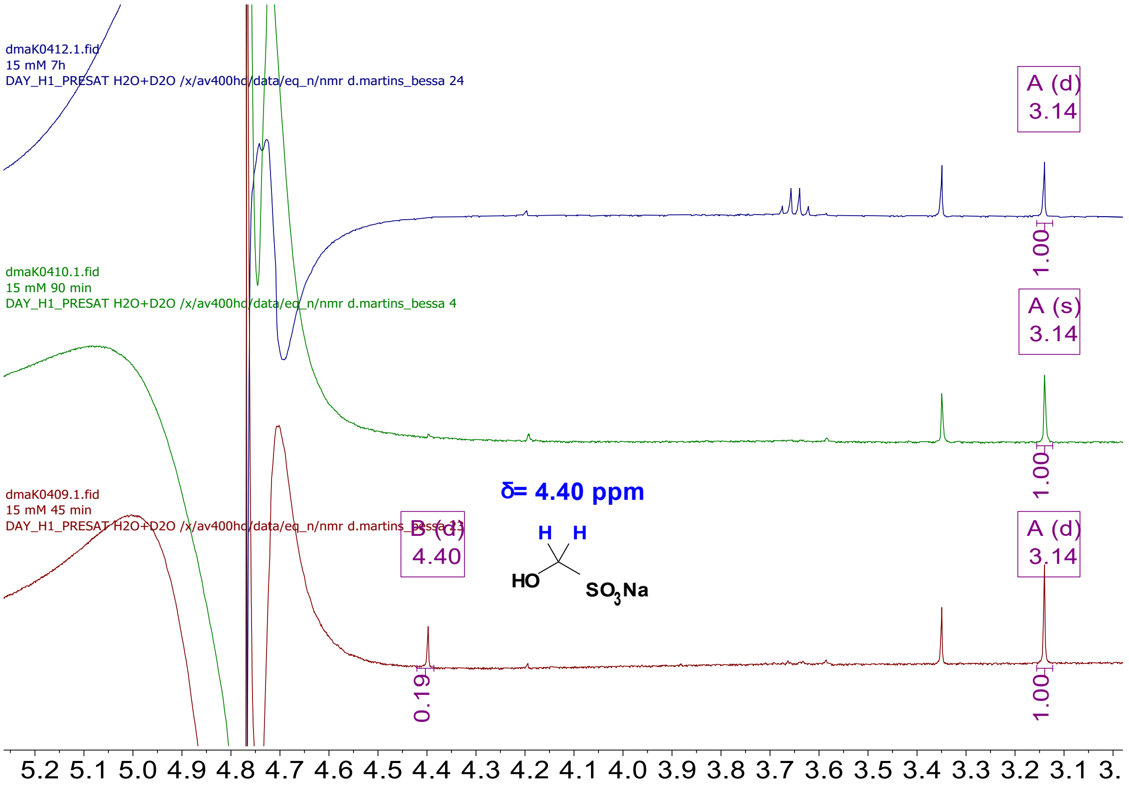

Figure S15. Stack of ^{1}H NMR spectra of the formose reaction after 45 min (red), 90 min (green) and 7 h (blue) at 15 mM of formaldehyde ($DMSO_2$: 3.14 ppm).

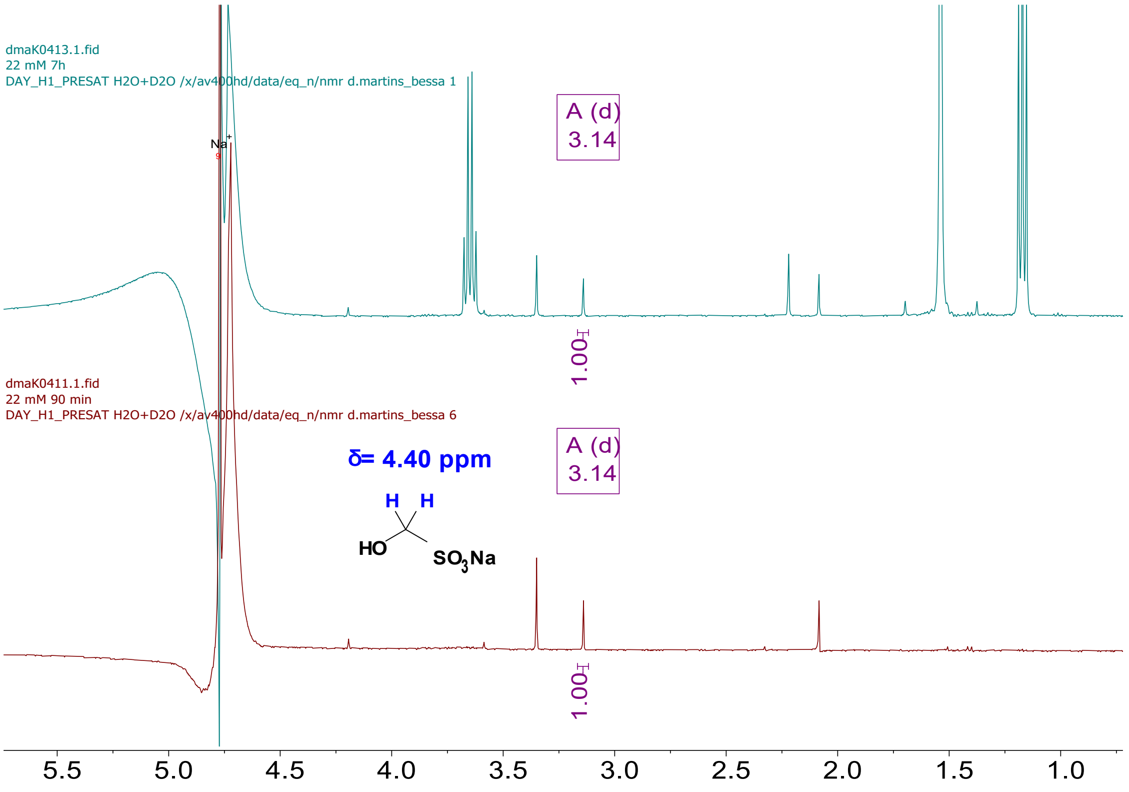

Figure S16. Stack of ^{1}H NMR spectra of the formose reaction after 90 min (red) and 7 h (blue) at 22 mM of formaldehyde ($DMSO_2$: 3.14 ppm).

3.6 Impact of the electrolyte

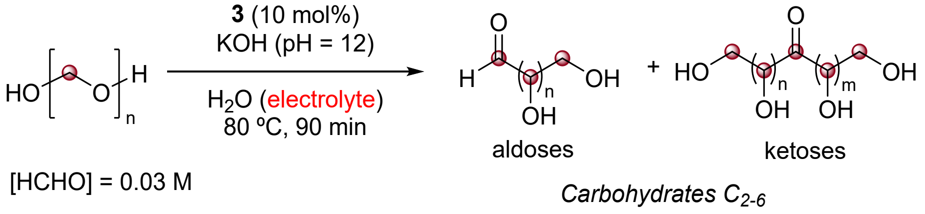

Electrolyte	Concentration (M)
K_2HPO_4	0.625
K_2CO_3	0.625
KCl	1.3

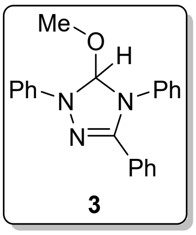

Scheme S6. Exploration of the electrolyte impact on the formose reaction.

In three different experiments, K_2HPO_4 (359 mg, 2.063 mmol, 0.625 M), K_2CO_3 (285 mg, 2.063 mmol, 0.625 M) and KCl (320 mg, 4.290 mmol, 1.3 M) were added before the formose reaction. **Figure S17** shows a stack of the three chromatograms and **Table S7** the calculated yields.

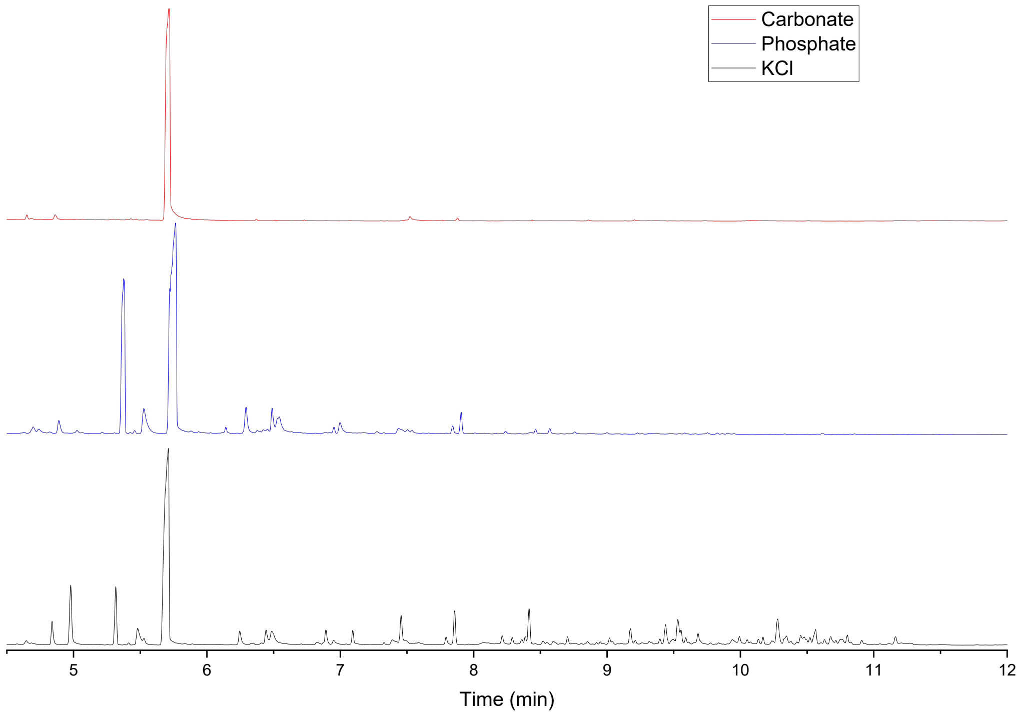

Figure S17. Chromatograms of the formose reaction in presence of 0.625 M of K_2CO_3, 0.625 M of K_2HPO_4 and 1.3 M of KCl (top to bottom).

Table S7. Chemical yields of the formose reaction in presence of different potassium salts.

Electrolyte	C_2 %	C_3 %	C_4 %	C_5 %	C_6 %	Total %
K_2CO_3	0	0	0	0	0	0
K_2HPO_4	0	0	0	0	0	0
KCl	0	0	0	23	23	46

In three different experiments, K_2HPO_4 (17.4 mg, 0.1 mmol, 1 equiv.), K_2HPO_4 (359 mg, 2.063 mmol, 0.625 M) and K_2CO_3 (285 mg, 2.063 mmol, 0.625 M) were added after the derivatization. **Figure S18** shows a stack of the three chromatograms and **Table S8** the calculated yields.

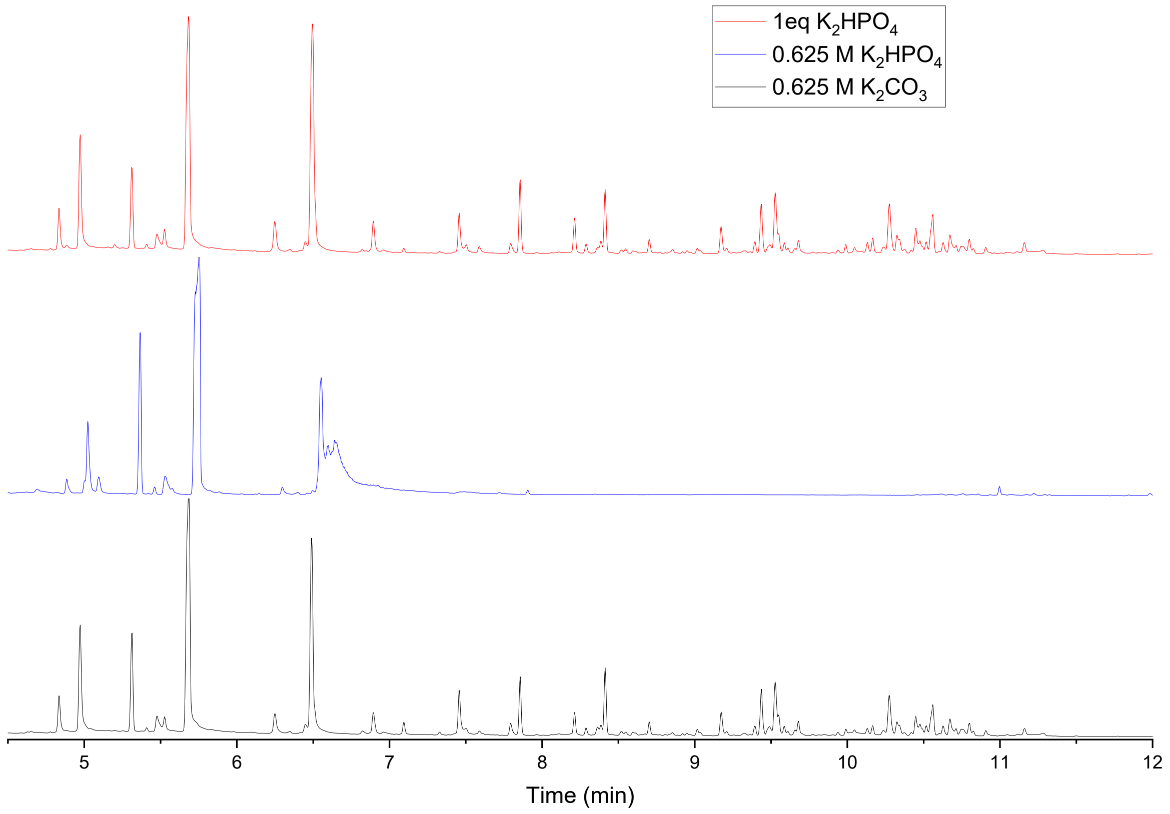

Figure S18. Chromatograms of the formose reaction in presence of 1 equiv. and 0.625 M of K_2HPO_4 and 0.625 M of K_2CO_3 added after the derivatization step (top to bottom).

Table S8. Chemical yields of the formose reaction in presence of different potassium salts added after the derivatization.

Electrolyte	Nb equiv. to HCHO	C_2 %	C_3 %	C_4 %	C_5 %	C_6 %	Total %
K_2HPO_4	1	0	0	0	19	18	37
K_2HPO_4	20	0	0	0	0	0	0
K_2CO_3	20	0	0	0	19	17	36

4. Formose reaction with commercial ^{13}C labelled paraformaldehyde

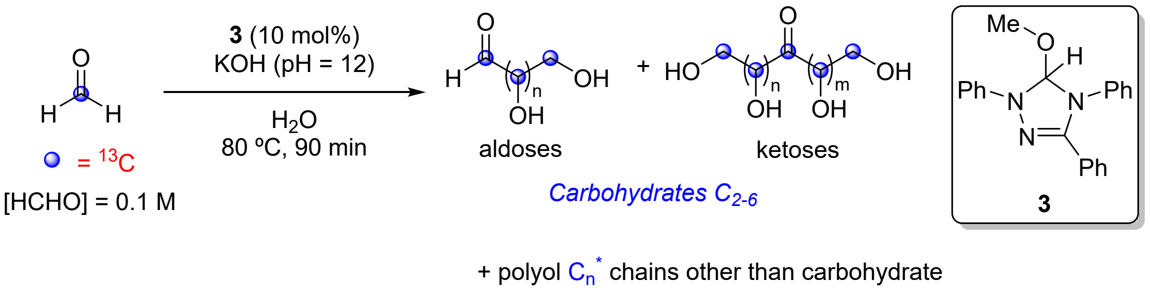

Scheme S7. Formose reaction using H^{13}CHO with catalyst **3**.

A 20 wt% aqueous solution of H^{13}CHO (50 µL, 99 atom% ^{13}C) and **3** (11.0 mg, 0.03 mmol) were mixed with 3 mL of solution A in a Pyrex tube (**Scheme S7**). The mixture was stirred at 80°C for 90 min and then the characterization of $^{13}C_{2\text{-}6}$ carbohydrates was carried out. In these conditions, the concentration [H^{13}CHO] = 0.1 M was chosen to ensure the detection of $^{13}C_{2\text{-}6}$ carbohydrates and not only $^{13}C_{5\text{-}6}$ carbohydrates that would have been produced at 0.03 M. **Figure S19** shows the recorded chromatogram stacked with the chromatogram obtained with commercial paraformaldehyde $(H^{12}CHO)_n$ at 0.03 M. The reaction with $(H^{12}CHO)_n$ was stirred at 80°C for 90 min with 10 mol% of **3** following the general procedure. This experiment further confirmed the attribution of the retention peaks to carbohydrates arising from the oligomerization of formaldehyde, and enabled also the attribution of retention peaks to polyoxygenated C_n* chains other than carbohydrates. **Table S9** shows the fragmentation data for the C_n carbohydrates and also for polyoxygenated C_n* chains other than carbohydrates with H^{12}CHO and H^{13}CHO to attribute the minimum number of carbon atoms in the C_n* chains.

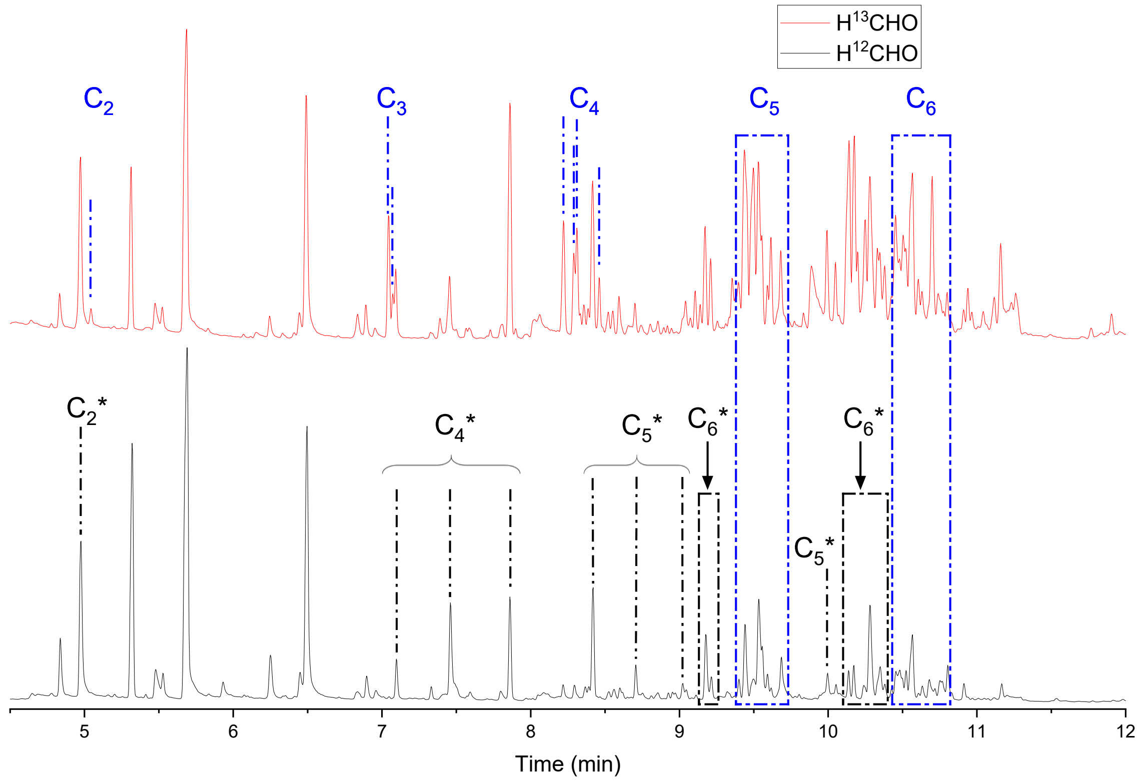

Figure S19. Stack of chromatograms of the reaction using H^{13}CHO and H^{12}CHO and identification of C_n carbohydrates and polyoxygenated C_n* chains other than carbohydrates.

Table S9. Comparison of the main fragments from MS with $H^{13}CHO$ and $H^{12}CHO$. Identification of C_n carbohydrates and polyoxygenated C_n* chains other than carbohydrates.

RT (min)	m/z fragments ($H^{13}CHO$)	m/z fragments ($H^{12}CHO$)	Attribution
4.97	207; 178	205; 177	C_2*
5.04	221; 206; 132	Table S2	C_2 carbohydrate
7.04-7.07	324; 309; 234; 220; 192; 104	Table S2	C_3 carbohydrate
7.10	339; 324; 249; 235; 116	335; 320; 245; 232; 114	C_4*
7.46	325; 222; 132; 104	321; 219; 129; 103	C_4*
7.86	399; 310, 220; 104	395; 307; 217; 103	C_4*
8.22-8.46	427; 412; 323; 221; 207	Table S2	C_4 carbohydrate
8.41	442; 427; 338; 263	437; 422; 334; 258	C_5*
8.55-8.59	442; 427; 338; 236; 207	-	
8.70	428; 397; 325; 294; 206; 132	423; 393; 321; 292; 203; 129	C_5*
9.02	428; 339; 249; 235	423; 335; 245; 231	C_5*
9.04	397; 352; 292	-	
9.11-9.14	354; 325; 221; 104	-	
9.18	369; 309; 235; 104	363; 305; 231; 103	C_6*
9.21	353; 323; 310; 221; 132; 104	348; 319; 307; 219; 103	C_6*
9.35	443; 368; 353; 264; 207	-	C_6*
9.40-9.68	530; 515; 440; 426; 310; 220	Table S2	C_5 carbohydrate
10.00	444; 426; 310; 264; 220	439; 421; 307; 259; 217	C_5*
10.14-10.37	546; 457; 324; 234; 104	540; 452; 321; 231; 103	C_6*
10.45-10.80	633; 618; 529; 426; 310; 220	Table S2	C_6 carbohydrate
10.91; 11.04-11.16	649; 560; 542; 323; 234; 220	-	$C_{n>7}$*
10.94	544; 454; 310; 220; 104	-	
10.97	649; 442; 324; 310; 220; 104	-	$C_{n>7}$*
11.23	664; 649; 574; 560; 470; 323; 220; 207; 192; 161	-	$C_{n>7}$*

We have tried to attribute the polyoxygenated C_n* chains other than carbohydrates more precisely than only the chain length (**Table S9**). **Table S10** shows these attributions that are only hypotheses, which should be confirmed by complementary analyses. The following EI-MS principles were considered for the attribution:

- The electron ionization (EI) source generates radical cations M^{+*} as molecular ions;
- The EI source is a hard ionization process and molecular ions can be unobservable or with a low intensity;
- The abstracted electron follows the relative orbital energies ($n > \pi > \sigma$). As a consequence, the electron is more easily abstracted from a lone pair;

- During the fragmentation, neutral radical or small molecules can be lost observing only cations (F^+) or radical cations (F^{+*});
- $HO\text{-}Si(CH_3)_3$ (m/z = 90) is commonly lost from trimethylsilyl C_n* compounds;
- No loss of H^- is not considered because unlikely with EI;
- Classical polyoxygenated C_n* families considered for the attribution were i) deoxy sugars (–OH groups replaced by –H); ii) carboxylic acids and iii) polyalcohols;
- Carboxylic acids do not form oximes due to its lower carbonyl reactivity;
- Nitrogen rule: for the molecular ions, m/z is an odd number if the molecule features a nitrogen atom (carbohydrates or deoxy sugars), otherwise is even (carboxylic acids and polyalcohols);
- $^{13}C_6$-carbohydrates; $^{13}C_6$-carboxylic acids; $^{13}C_6$-deoxy sugars and $^{13}C_6$-polyalcohols give molecular ions of m/z = 633; 634; 539 and 614 implying that peaks with m/z > 634 are not $^{13}C_6$* compounds.

For deoxy-sugars only aldo isomers were proposed for simplicity.

Table S10. Proposed structures for the polyoxygenated C_n* chains other than carbohydrates (underlined compounds were observed only in the $H^{13}CHO$ experiment).

Retention times	Proposed M^{+*} structures	Observed fragments
4.97	*C_2* acetic acid*<br>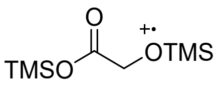<br>m/z = 220	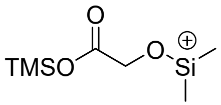<br>m/z = 205
7.10	*C_4* deoxy sugar*<br>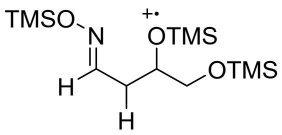<br>m/z = 335	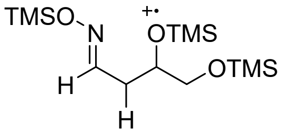<br>m/z = 335<br>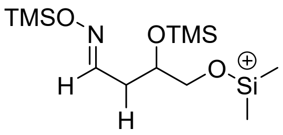<br>m/z = 320<br>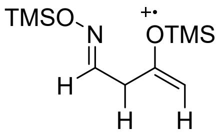<br>m/z = 245<br>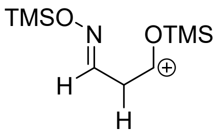<br>m/z = 232
7.46	*C_4* carboxylic acid*<br>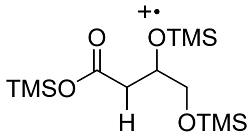<br>m/z = 336	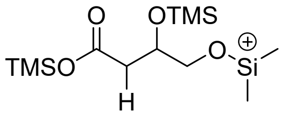<br>m/z = 321<br>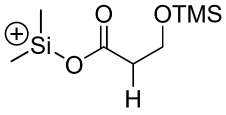<br>m/z = 219<br>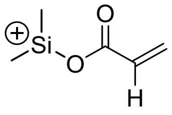<br>m/z = 129<br>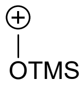<br>m/z = 103
7.86	*C_4* polyalcohol*<br>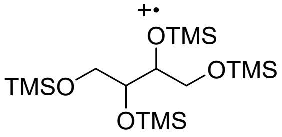<br>m/z = 410	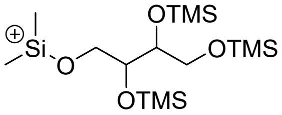<br>m/z = 395<br>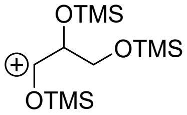<br>m/z = 307<br>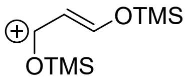<br>m/z = 217<br>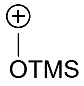<br>m/z = 103

8.41; 8.55-8.59	*C_5* deoxy sugar* 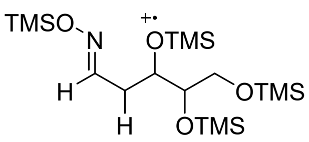 m/z = 437	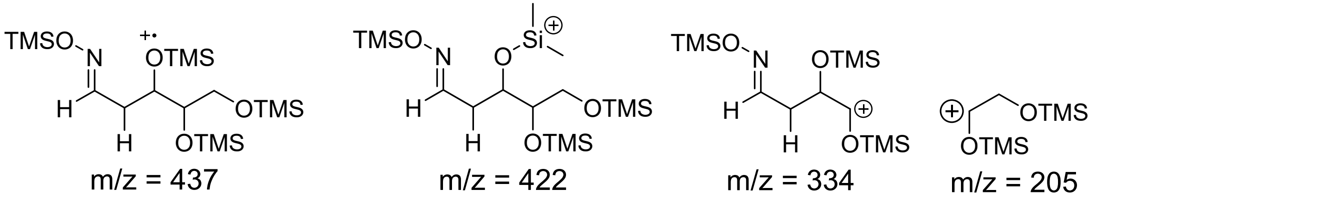
8.70	*C_5* carboxylic acid* 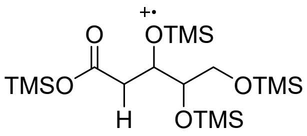 m/z = 438	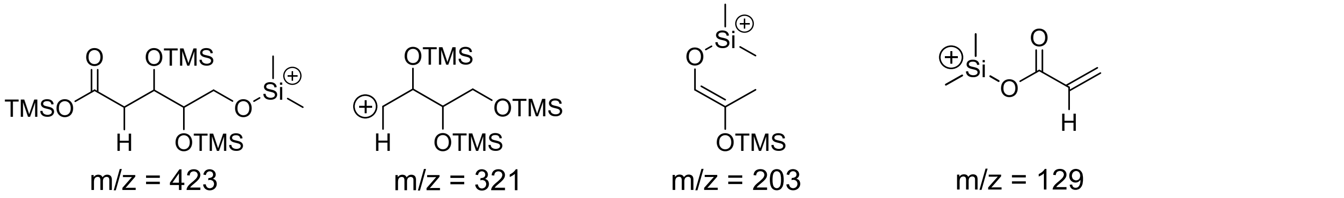
9.02	*C_5* carboxylic acid* 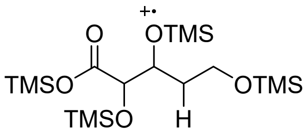 m/z = 438	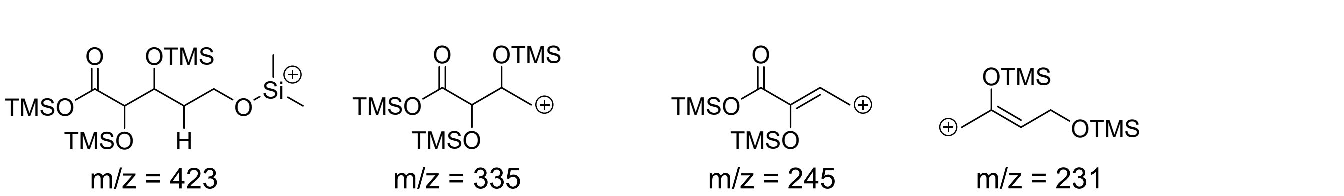
9.04	Not attributed	
9.11-9.14	*C_5* carboxylic acid* 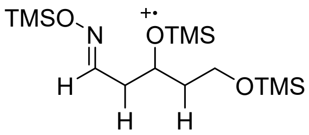 m/z = 349	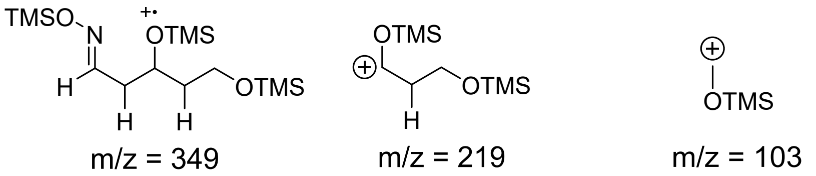
9.18	Not attributed	

9.21	C_6* *carboxylic acid* 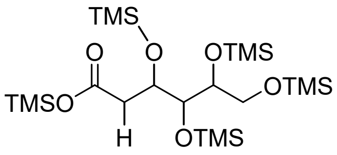 m/z = 540	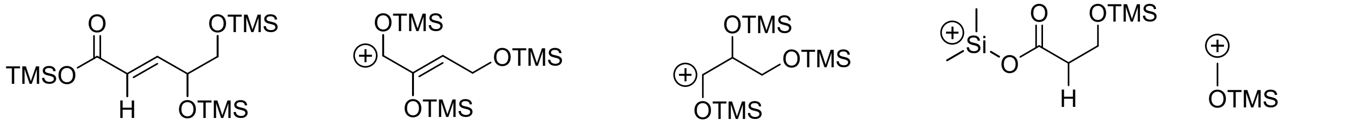 m/z = 348; m/z = 319; m/z = 307; m/z = 219; m/z = 103
9.35	C_6* *carboxylic acid* 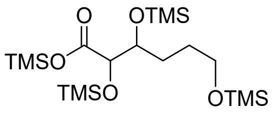 m/z = 452	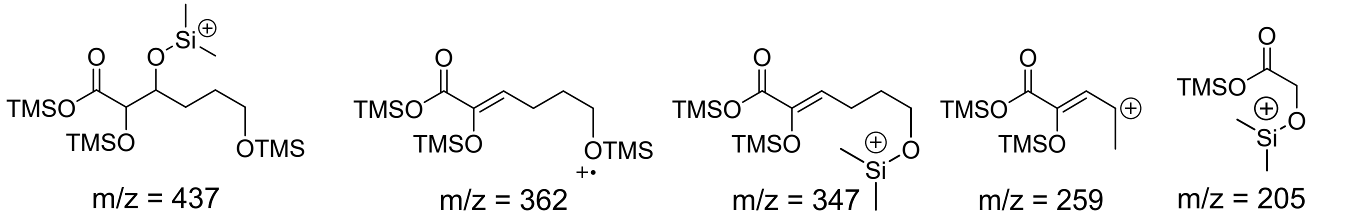 m/z = 437; m/z = 362; m/z = 347; m/z = 259; m/z = 205
10.00	Not attributed	
10.14-10.37	C_6* *carboxylic acid* 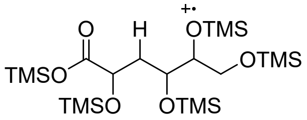 m/z = 540	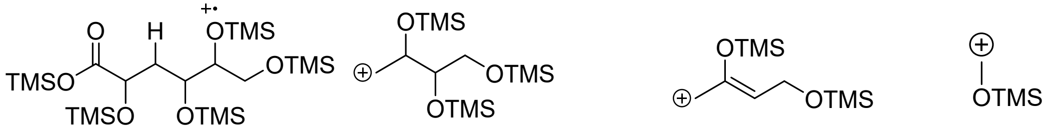 m/z = 540; m/z = 321; m/z = 231; m/z = 103
10.91; 11.04-11.16	$C_{n>7}$*	
10.94	C_6* *carboxylic acid* 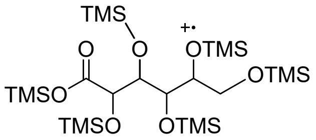 m/z = 628	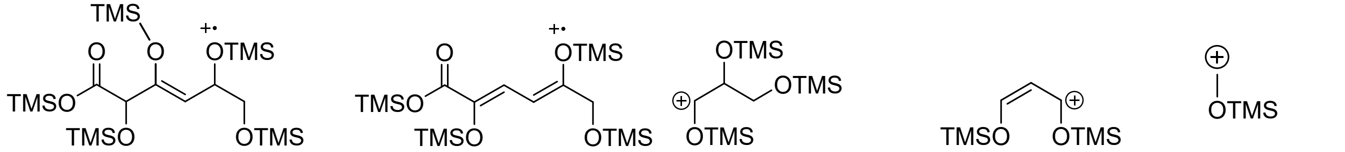 m/z = 538; m/z = 448; m/z = 307; m/z = 217; m/z = 103
10.97	$C_{n>7}$*	
11.23	$C_{n>7}$*	

5. One-pot-two-step conversion of CO into C_{5-6} carbohydrates

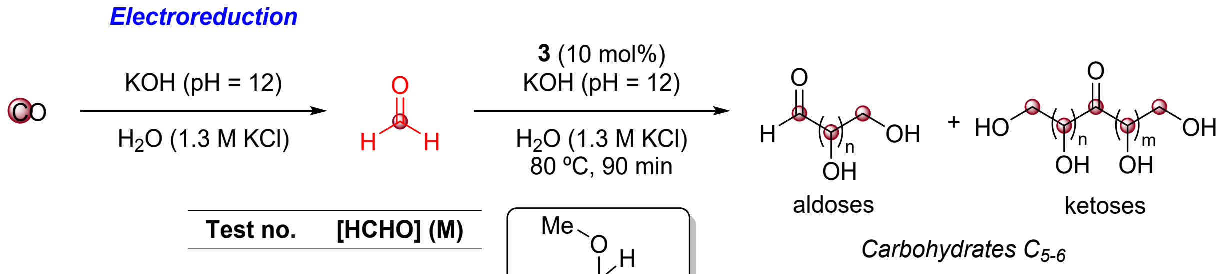

Test no.	[HCHO] (M)
**1**	0.029
**2**	0.027

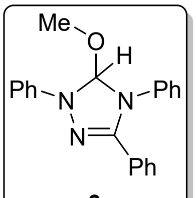

3

Scheme S8. One-pot two-step conversion of CO into carbohydrates.

As described in Section 2.3, four consecutive CPEs were conducted on the same solution for a total of 35h to accumulate HCHO up to *ca.* 30 mM (**Scheme S8**).

A solution of **3** (7 mg, 0.021 mmol for test 1; 9 mg, 0.027 mmol for test 2) in THF (1 mL) was prepared. This solution of **3** (410 µL, 0.009 mmol for test 1; 50 µL, 0.0014 mmol for test 2) was introduced in a Pyrex tube and the THF was evaporated under vacuum. Then, formaldehyde solution obtained by the CO electroreduction (3 mL, 0.087 mmol for test 1; 0.5 mL, 0.014 mmol for test 2) was added to the Pyrex tube and the formose reaction was carried out.

Figure S20-21 shows the chromatogram obtained with 29 mM and 27 mM, respectively. **Table S10** indicate the yield calculated for both reactions.

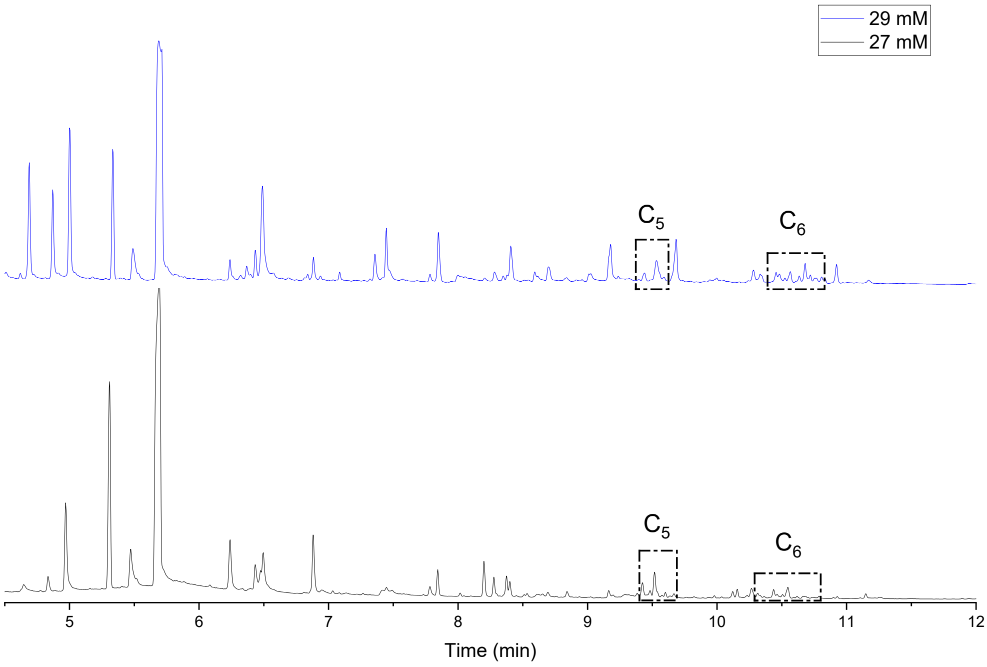

Figure S20. Chromatograms obtained using the electroreduction sourced formaldehyde at 29 mM (blue) and 27 mM (black).

Table S11. Chemical yields of the formose reaction carried out with formaldehyde obtained upon CO electroreduction.

[HCHO]	C_2 %	C_3 %	C_4 %	C_5 %	C_6 %	Total %
29 mM	0	0	0	12	9	21
27 mM	0	0	0	18	4	22

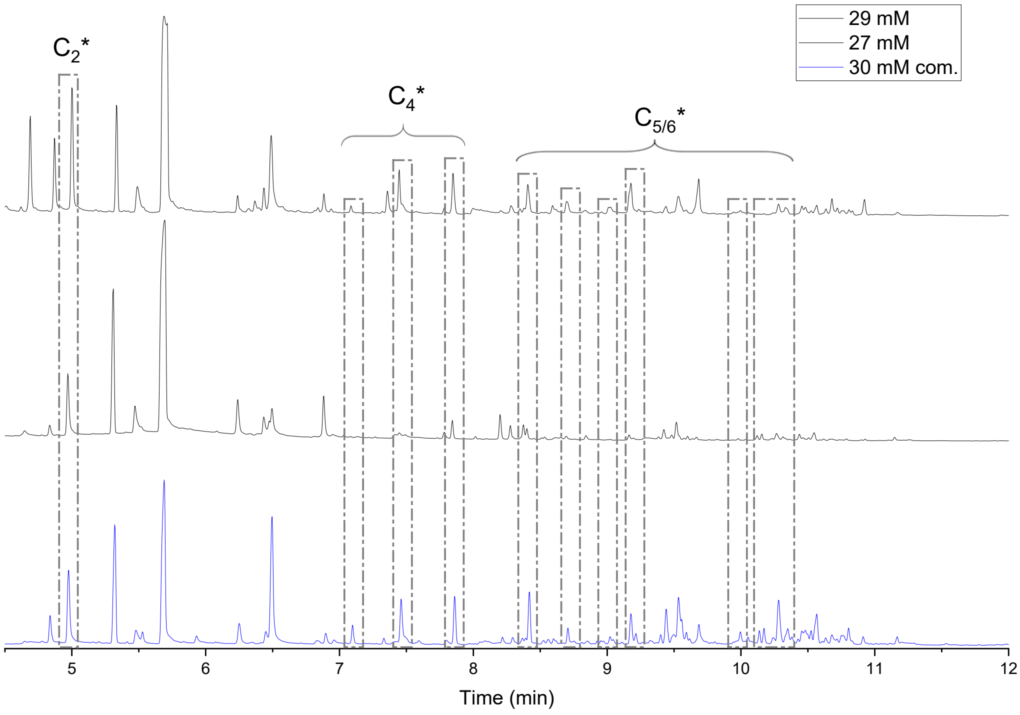

Figure S21. Identification of C_n* polyol chains other than carbohydrates based on **Table S9**.

6. Figures of the electroreduction of CO into formaldehyde

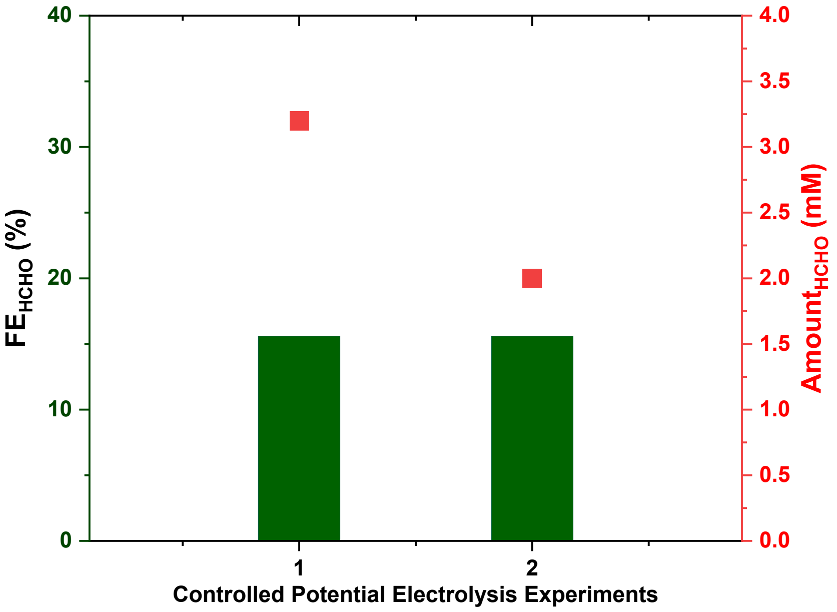

Figure S22. Faradaic efficiencies (FE) and molar amounts of formaldehyde in 2 h controlled potential electrolysis in a three-compartment closed cell, run 1 and 2 using the same 1x1 cm^2 carbon paper electrode drop-casted with CoPc/MWCNT ink at -0.65 V *vs.* RHE at pH 12 (T = 10°C, 85% IR compensation) showing 15. 6% FE_{HCHO} (green bars) in both cases and 3.2 mM and 2 mM amounts (red squares) of HCHO, respectively; see **Figures S23-24** for their respective NMR spectrum.

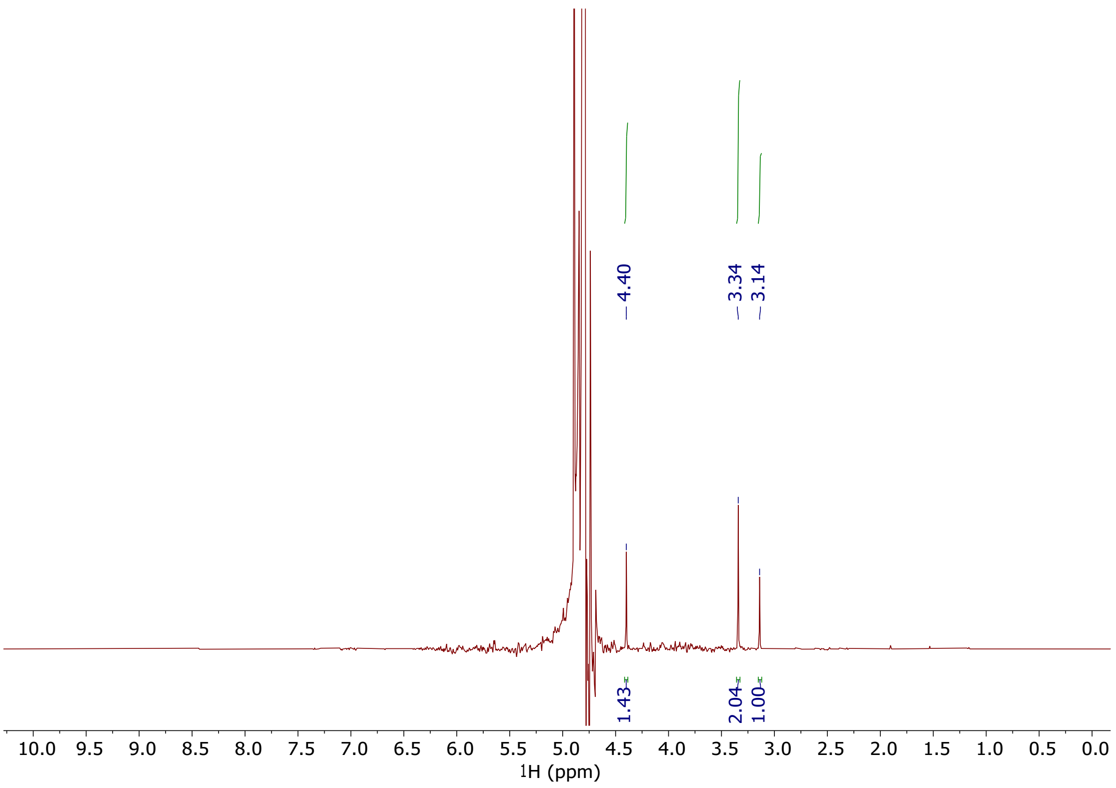

Figure S23. ^1H NMR after CPE (run 1) using 1x1 cm^2 carbon paper electrode drop-casted with CoPc/MWCNT ink in the presence of carbon monoxide at -0.650 V *vs.* RHE at pH 12 (T = 10 °C, 85% IR compensation); δ 8.44 ($HCOO^-$), 4.4 (hydroxy methane sulfonate), 3.34 (CH_3OH), 3.14 ($DMSO_2$)

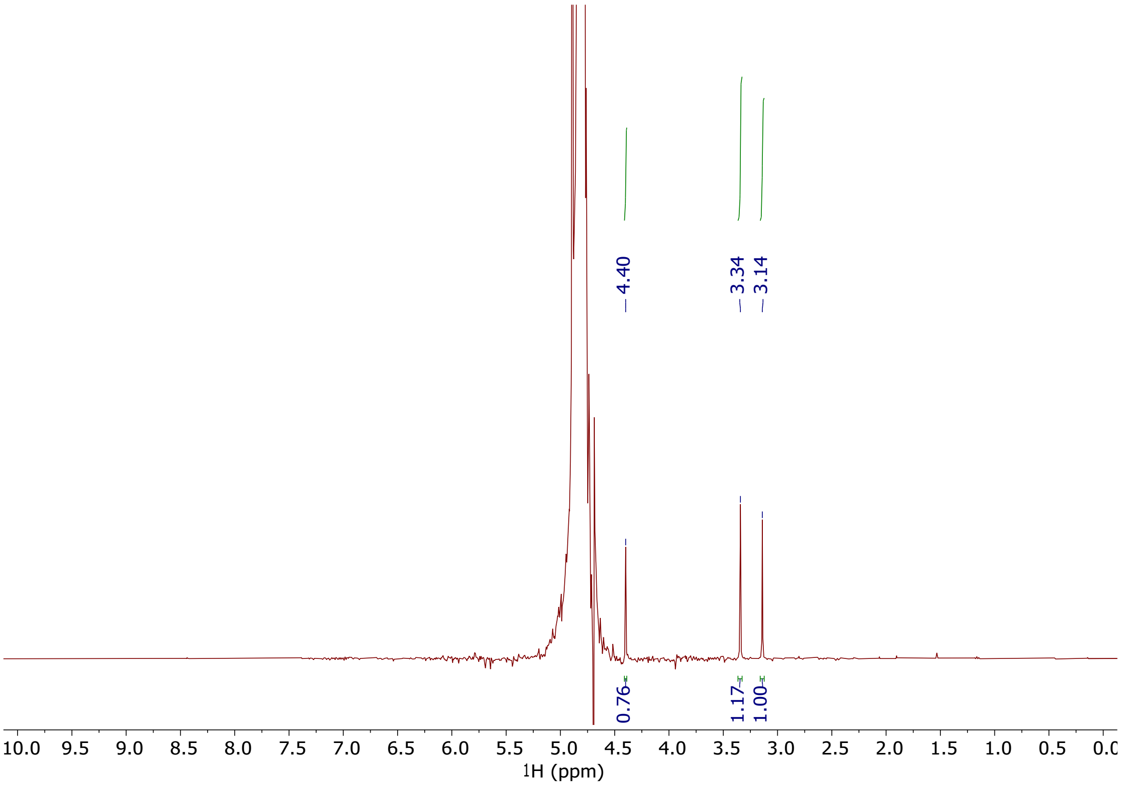

Figure S24. ^{1}H NMR after CPE (run 2) using used after run 1, 1x1 cm^2 carbon paper electrode drop-casted with CoPc/MWCNT ink in the presence of carbon monoxide at -0.650 V *vs.* RHE at pH 12 (T = 10°C, 85% IR compensation); δ 8.44 ($HCOO^-$), 4.4 (hydroxy methane sulfonate), 3.34 (CH_3OH), 3.14 ($DMSO_2$)

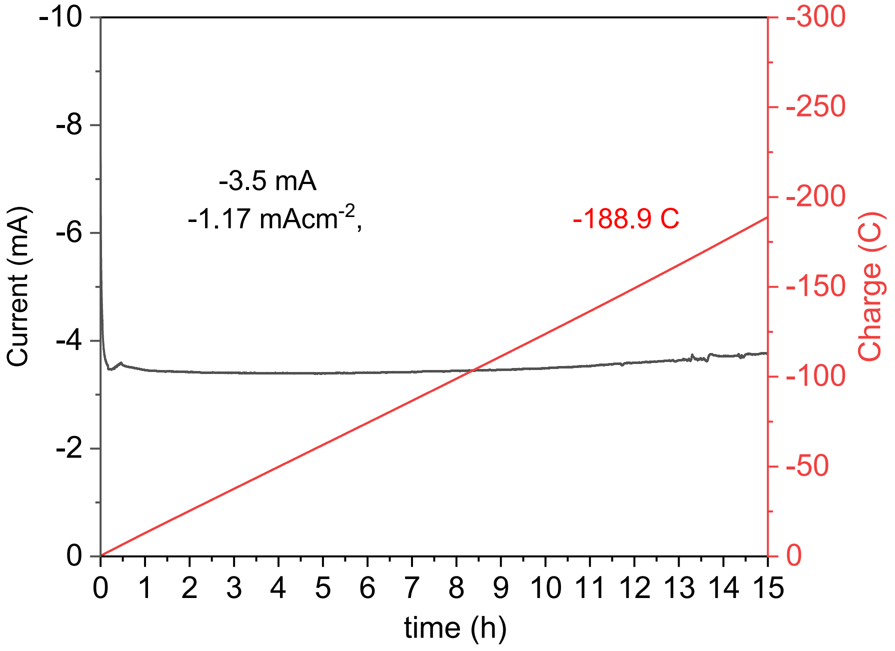

Figure S25. 15 h CPE in the presence of carbon monoxide at -0.650 V *vs.* RHE at pH 12 using phosphate buffer (T = 10°C, 85% IR compensation), showing a stable current density value of -1.17 mA/cm^2 (black line) and charge value of -188.9 C (red line)

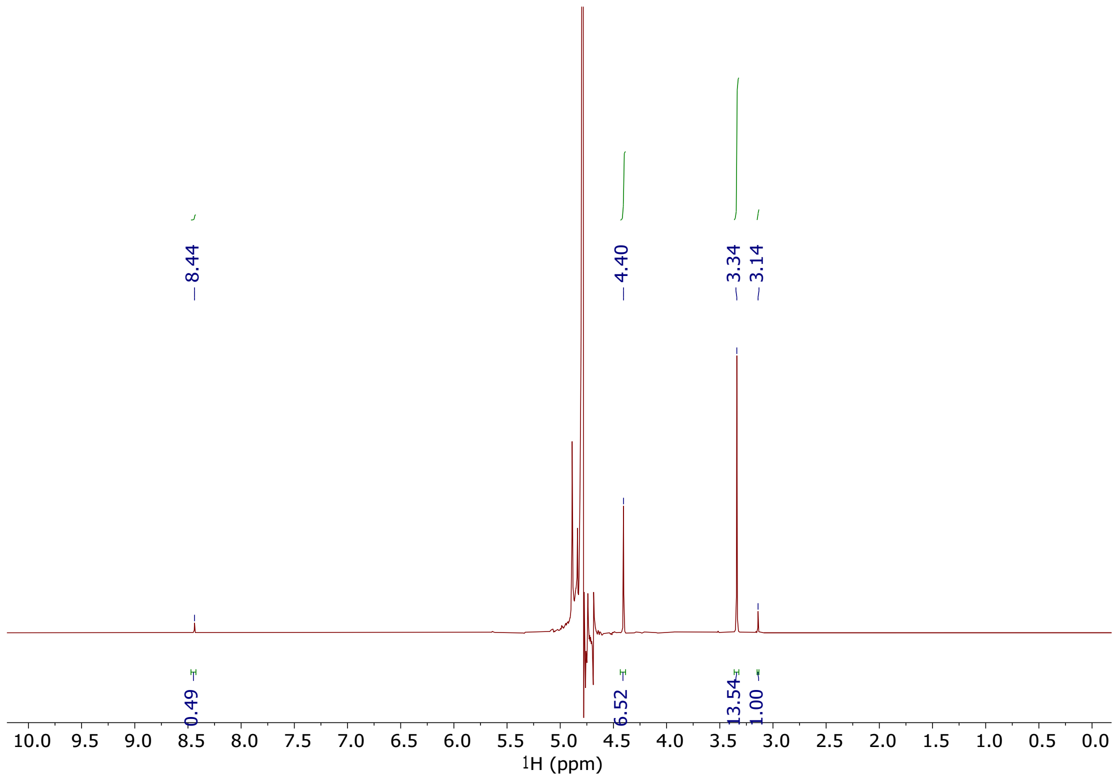

Figure S26. ^{1}H NMR after fifteen hours of CPE in the presence of carbon monoxide at -0.650 V *vs.* RHE at pH 12 using phosphate buffer (T = 10°C, 85% IR compensation); δ 8.44 ($HCOO^-$), 4.4 (hydroxy methane sulfonate), 3.34 (CH_3OH), 3.14 ($DMSO_2$)

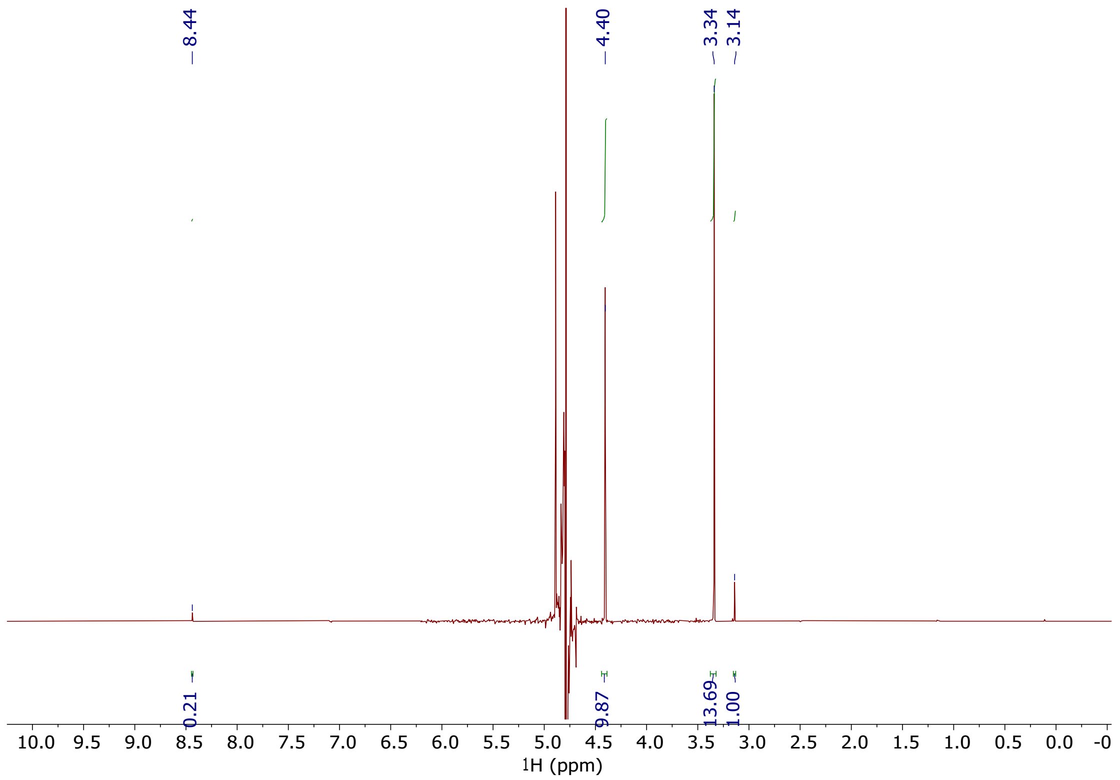

Figure S27. ^{1}H NMR after thirty hours CPE in the presence of carbon monoxide at -0.650 V vs. RHE at pH 12 using phosphate buffer (T = 10°C, 85% IR compensation); δ 8.44 ($HCOO^-$), 4.4 (hydroxy methane sulfonate), 3.34 (CH_3OH), 3.14 ($DMSO_2$)

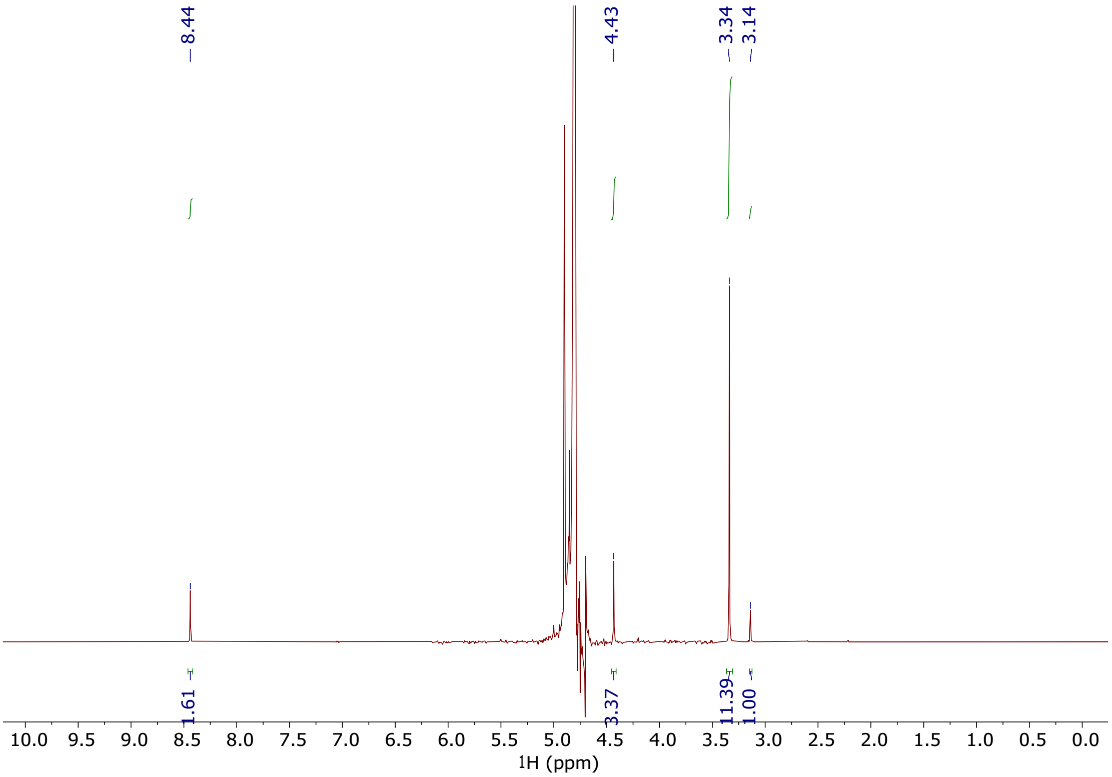

Figure S28. ^{1}H NMR after 42 h CPE in the presence of carbon monoxide at -0.650 V vs. RHE at pH 12 using phosphate buffer (T = 10°C, 85% IR compensation); δ 8.44 ($HCOO^-$), 4.4 (hydroxy methane sulfonate), 3.34 (CH_3OH), 3.14 ($DMSO_2$)

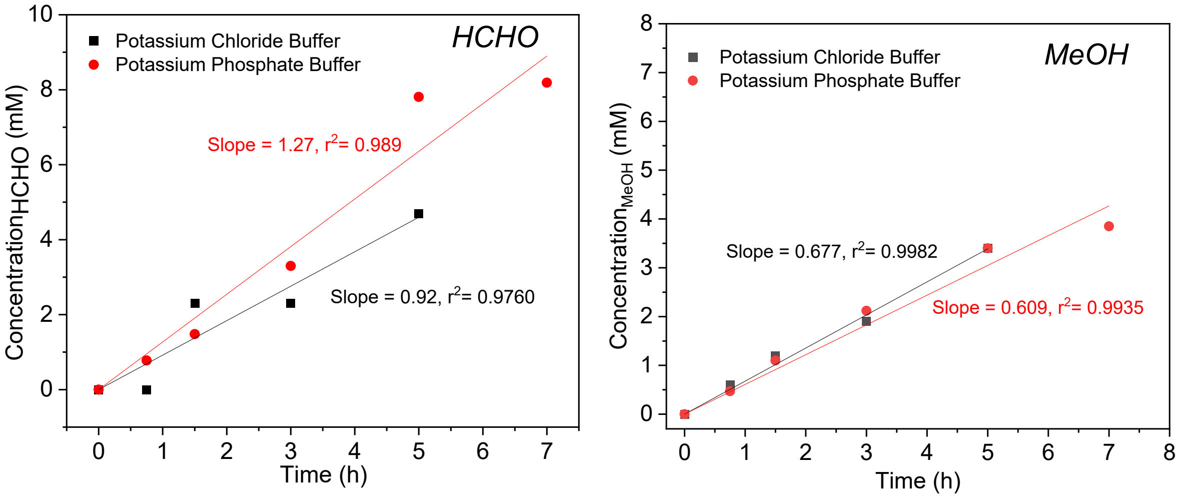

Figure S29. Comparison of CPE in the presence of carbon monoxide at -0.650 V vs. RHE at pH 12 using phosphate buffer (red dots and line) and KCl buffer (black dots and line) (T = 10°C, 85% IR compensation). Note that the rate of formation of HCHO is slightly lower in KCl buffer than in phosphate buffer. However, methanol production was almost the same.

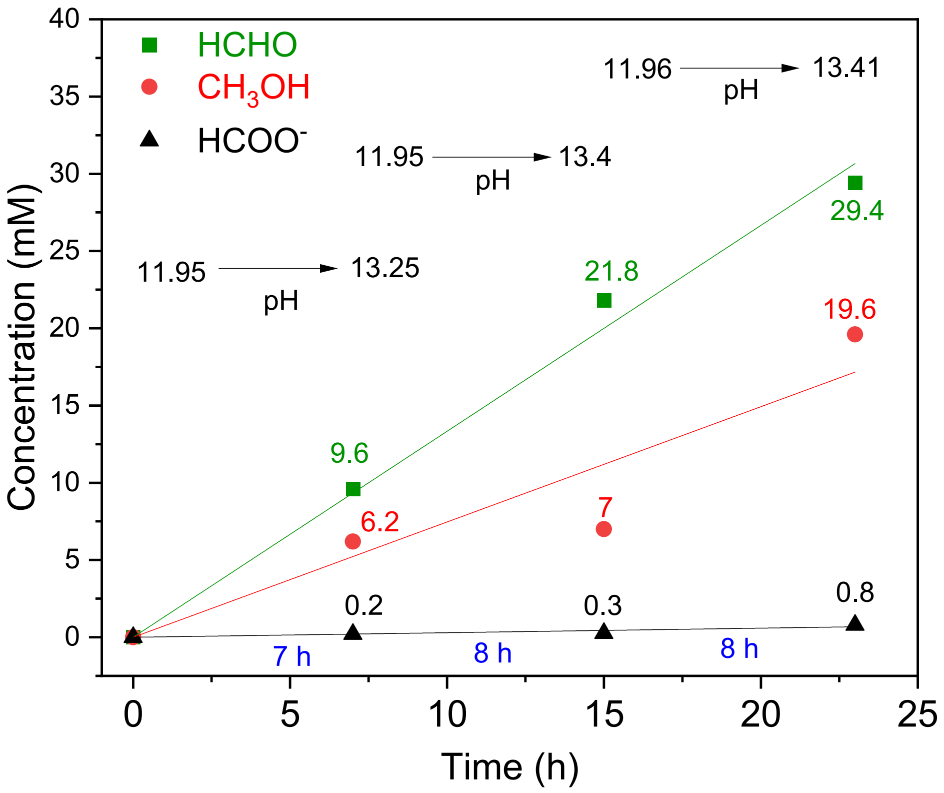

Figure S30. Time-dependent production of HCHO using KCl buffer (using 12.5 mL solution as electrolyte from 20 mL prepared by mixing 1 M KOH and 4 mL 5 M HCl to reach pH 12); green line = HCHO concentration in mM; red line = CH_3OH concentration in mM; blue line = $HCOO^-$ concentration in mM; 7 h, 8 h, 8 h in blue represent time of consecutive CPE experiments; black arrow with values show initial and final pH values; adjusted pH 13.25 to 11.95 and 13.4 to 11.96 using 5M HCl and 1M KOH followed by 15 min argon and 20 min CO bubbling of electrolyte solution (CPE = -0.65 V *vs.* RHE, pH = 12, T = 10°C, 85% IR compensation).

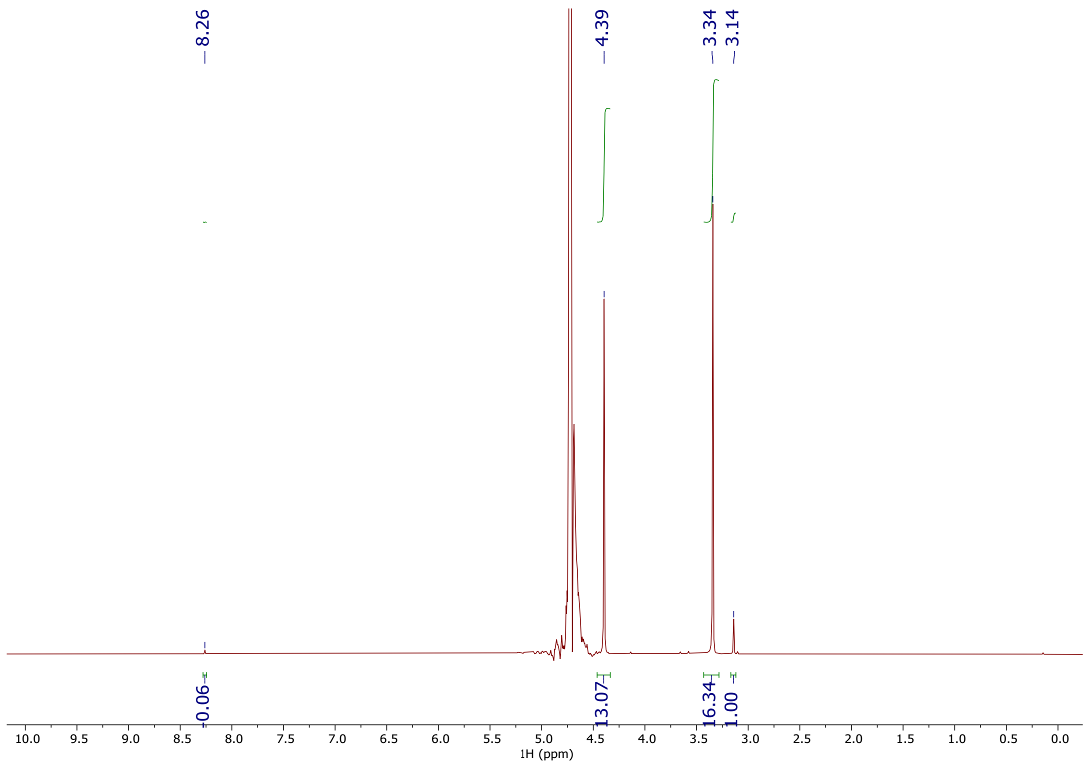

Figure S31. ^{1}H NMR of the electrolyte after 7 h + 8 h + 8 h CPEs using KCl buffer (12.5 mL solution as electrolyte from 20 mL prepared by mixing 1 M KOH and 4 mL 5 M HCl to reach pH 12); the very high integration value of 13.07, compared to internal standard ($DMSO_2$) one of 16.34 at chemical shift 4.4 ppm, demonstrates the very large amount of electrochemically produced HCHO.

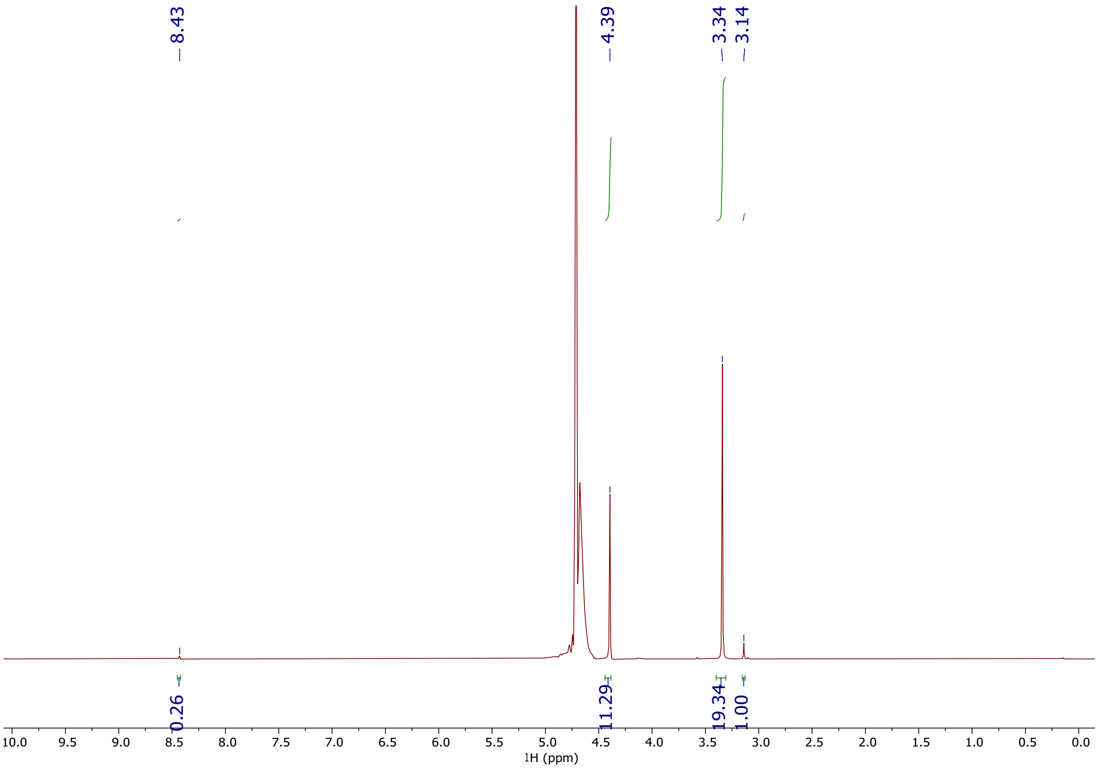

Figure S32. ^{1}H NMR of the electrolyte after 7 h + 8 h + 8 h + 8 h CPEs using KCl buffer (12.5 mL solution as electrolyte from 20 mL prepared by mixing 1 M KOH and 4 mL 5 M HCl to reach pH 12); the very high integration value of 11.29, compared to internal standard ($DMSO_2$) one at chemical shift 4.4 ppm, demonstrates the very large amount of electrochemically produced HCHO.

Table S12. Optimization of controlled potential electrolysis conditions for higher formaldehyde production.

Serial no.	Variable parameter(s)	Time (h)	Volume of cell (mL)	No. of electrode	Carbon electrode area (cm^2)	[HCHO]
**1**	time$^{\alpha}$	0.5	5	1	1x1	1.2 mM
**2**	time$^{\alpha}$	2	5	1	1x1	4 mM
**3**	time and balloon$^{\alpha}$	5	5	1	1x1	4.2 mM
**4**	time, surface, catalyst amt. and volume of cell	10	12.5	1	1.5x2	9.6 mM
**5***	time	12	12.5	1	1.5x2	14.6 mM
**6***	time	15	12.5	1	1.5x2	17.4 mM
**7**	time	14	12.5	2	1.5x2	13.9 mM
**8***	time	30	12.5	1	1.5x2	23.9 mM

*Surface, catalyst amount and volume of cell were kept as condition **4**
